# The impact of digital art-making on anxiety: a feasibility study

**DOI:** 10.3389/fpsyg.2025.1620583

**Published:** 2025-09-15

**Authors:** Laura M. H. Gallo, Vincent Giampietro, Minu Choi

**Affiliations:** ^1^Department of Neuroimaging, Institute of Psychiatry, Psychology and Neuroscience, King’s College London, London, United Kingdom; ^2^Department of Psychology, Goldsmiths, University of London, London, United Kingdom

**Keywords:** neuroesthetics, mental wellbeing, digital art making, state–trait anxiety inventory, esthetic responsiveness, art interventions

## Abstract

Extensive research shows art-making reduces stress and activates the brain’s reward system, yet few studies explore remote digital art interventions. This feasibility study examines whether a remote digital art-making intervention can reduce anxiety. It offers preliminary insights into the potential of online art tools to broaden access to creative expression. Participants engaged in an eight-week program using the Mindful of Art platform, which enables digital art-making without technical skills. Each week, they completed a themed task designed to encourage creativity and reduce self-criticism, along with surveys measuring anxiety, flow, and affect. At registration, we collected demographic data, artistic interest, self-perceived creativity, and trait anxiety. Trait anxiety was reassessed after 8 weeks, regardless of participation level. Results suggest digital art-making can help reduce anxiety, especially in highly anxious individuals. Effects were independent of artistic interest or self-perceived creativity. This suggests self-guided digital art-making could be a scalable, affordable complement to existing mental health services. While intervention frequency did not predict outcomes, our findings align with prior research suggesting art based interventions support mental wellbeing, and highlight the potential of digital art-making as an accessible, low skill self-help tool for anxiety management.

## Introduction

The COVID-19 pandemic led to both financial and mental health crises, with global rises in anxiety and depression (Roper and Turner, 2020; Tsamakis et al., 2021). Research suggests that engaging with the arts benefits mental wellbeing. While passive art consumption has some positive effects, active art creation offers additional advantages, such as self-expression, problem-solving, and a sense of accomplishment (Drake et al., 2024). It also fosters mindfulness, helping individuals stay present and disconnect from daily worries.

Systematic reviews indicate that creative art interventions alleviate anxiety and depression (Abbing et al., 2018; Bosman et al., 2021; Martin et al., 2018), including in high-stress groups like cancer patients (Bozcuk et al., 2017; Jalambadani and Borji, 2019; Radl et al., 2018), prisoners (Yu et al., 2016), and healthcare professionals (Huss and Sarid, 2014). Large-scale studies during the pandemic found that home-based arts engagement increased during lockdowns and declined as restrictions eased, suggesting people turned to the arts for stress relief (Bu et al., 2022; Bone et al., 2024).

Unfortunately, some may find developing art-making skills overwhelming and avoid participation. Digital art-making, which often requires only basic image editing skills, could be a more accessible alternative. While many studies compared online and in-person art engagement during and after the pandemic (Payne et al., 2024; Yang et al., 2022), research on digital art-making remains limited. This study focuses on visual digital art—which by definition is a non-atom based image made out of bits (Lin, 2005)—and assesses the feasibility of further research in this area.

The COVID-19 pandemic accelerated digital transformation, reshaping how we access services, study, receive news, and interact socially (Schilirò, 2020; Wade and Shan, 2020). As physical and digital spaces merge, it is not surprising that digital art engagement has risen (Enhuber, 2015; Trupp et al., 2023).

Recent studies suggest digital art viewing reduces negative mood and anxiety (Trupp et al., 2022; Cotter et al., 2023). Given these benefits, it is worth exploring whether digital art-making has a similar or greater impact. This question is especially relevant following Drake et al. (2024), who found art-making improved affect more than passive art viewing.

With mental health workforce shortages (Agyeman-Manu et al., 2023; Hoffmann et al., 2023) and only about half of users benefiting from online cognitive behavioral therapy (CBT)-based self-help tools (Prasad et al., 2023), accessible alternatives are needed. Digital art-making interventions could help those who find CBT or mindfulness apps unappealing or inefficient (Gallo et al., 2021). In a large study by Fancourt et al. (2020), most participants with depression, anxiety, or no mental health issues cited lack of skills, energy, or strength as barriers to art engagement. Digital art-making interventions can help overcome these barriers.

Visual art-making involves manipulating internal representations (Jankowicz, 1987) in pursuit of an artwork. Even experienced artists start with vague concepts, refining them through skill and persistence (Mace and Ward, 2002).

This is why we designed an online digital art course for beginners, starting with simple tasks that gradually introduce artistic composition concepts. Participants experiment with shapes, colors, and tones, building confidence over time.

By 2030, mental health issues, particularly depression and anxiety, are expected to be the leading global disease burden (Chisholm et al., 2016). Expanding access to support is critical, as most affected individuals can recover (Lancet, 2022). However, mental health services are not growing at the needed rate, and self-help tools with minimal therapist input can be as effective as therapist-led interventions (King et al., 2017)—though their effectiveness may vary, as higher anxiety is associated with decreased odds of improvement (Kunkle et al., 2021). To address this crisis, alternative first-line self-help tools are needed. This feasibility study explored whether further research into digital art-making interventions is warranted. Our hypotheses for this research were:

*Hypothesis* 1: Participants undertaking our course will benefit from a mood boost even if they don’t have previous art-making experience or have limited art knowledge.

*Hypothesis* 2: Participants who complete more weekly tasks will demonstrate stronger anxiety reduction effects.

*Hypothesis* 3: Participants’ flow and arousal will increase as the weeks progress and the art tasks get more complex.

*Hypothesis* 4: Participants with moderate anxiety will benefit the most from this intervention.

## Methods

This study has been reviewed and approved by the Research Ethics and Integrity Sub-Committee at Goldsmiths, University of London, which is committed to compliance with the Universities UK Research Integrity Concordat.

### Study design

This study employed a pre-post design to evaluate the effects of a digital art-making intervention on anxiety. We decided to follow the structure of a similar study conducted with medical students investigating whether art-making interventions (visual journaling in this case) could be used as a possible stress-reduction technique (Mercer et al., 2010). We used the STAI Y-2 to measure trait anxiety before and after the 8-week study to evaluate the intervention’s effects over time. For weekly measurements, we used the STAI Y-1 to capture state anxiety right after the art intervention had taken place. We also included weekly flow (Dispositional Flow Scale-2), and arousal and positivity (Affect Grid) post task surveys to investigate the potential effects of the intervention on these mental states.

### The digital art-making course

We developed an eight-week digital art-making course to examine its impact on anxiety. With no prior self-guided digital art studies to replicate, we modeled our design on mindfulness-based self-help interventions, which range from 10 days to 12 weeks. A systematic review found that 8 weeks was the most common duration among 83 mindfulness studies, showing a strong link between program length and effect size (Taylor et al., 2021). This duration also allowed us to assess whether the number of art sessions influenced outcomes.

Participants needed a computer with internet access for this self-driven course, completing tasks independently without supervision. Each week, they engaged in digital art-making and answered surveys measuring anxiety, flow, and affect. Responses remained anonymous. The estimated time commitment was 30–60 min per week including the survey time, similar to mindfulness interventions. Participants registered via an initial survey before starting their first digital art-making task.

Mindfulness courses use common themes to guide meditation (Barkhuizen et al., 2018). Inspired by this, we introduced weekly themes—acceptance, appreciation, attention, compassion, attunement, confidence, kindness, and self-compassion—to help participants overcome thought patterns that might hinder their creative journey.

The Mindful of Art platform^1^ allows users to create digital collages using fragments of professional artwork, enabling accessible art-making to all skill levels. Designed as a user-friendly self-help tool, it encourages creativity and original composition.

After each task, participants completed a weekly survey and had to wait a week before progressing. Initially designed as an eight-week course, the participation period was extended due to technical issues. Participants could pause and resume at any time. All received a final survey 8 weeks after their registration, regardless of task completion.

### Participants

**Figure 1:**
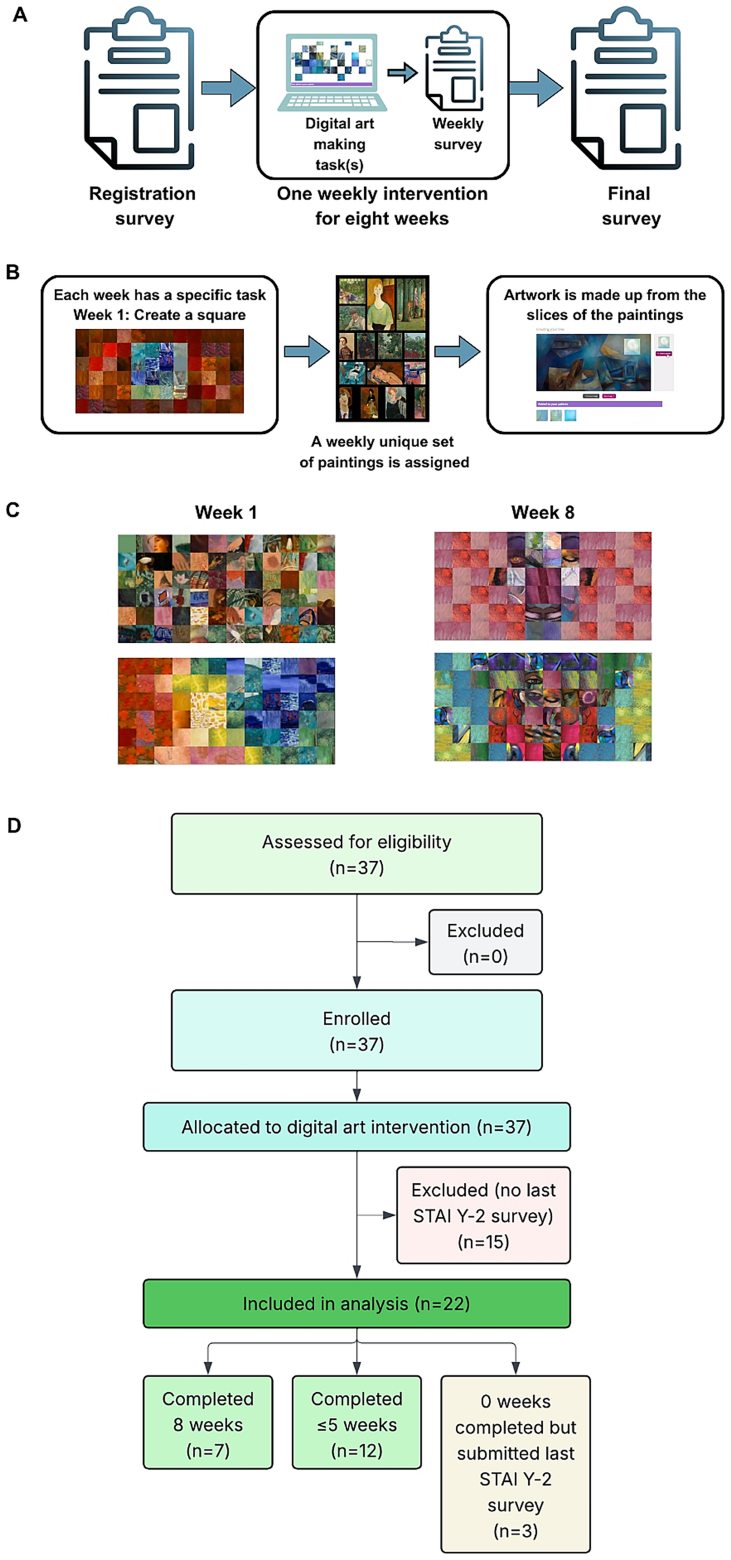
Study design, intervention, and participants’ artwork. **(A)** The study included an initial survey, weekly tasks followed by surveys, and a final survey 8 weeks later, regardless of task completion. **(B)** Artworks were digital mosaics created using square slices from professional paintings, with controls for luminance and rotation. **(C)** Weekly tasks increased in complexity, from creating a square (Week 1) to a self-portrait (Week 8). Examples from two participants show their progression in digital art-making. **(D)** CONSORT-style flow diagram illustrating participant progression through the 8-week digital art therapy course.

This feasibility study included a sample of 22 participants (13 women, 7 men, 1 non-binary, 1 prefer not to say; mean age = 36.86, SD = 7.03, age range = 19–72). Participants were invited to complete an 8-week digital art-making course and were asked to complete a survey before starting the course, surveys after finishing each weekly task and a final survey -after finishing the course. The final sample of 22 was derived from an initial sample of 37, but some participants were removed due to not completing any surveys apart from the initial one. A CONSORT-style flow diagram (see Figure 1D) summarizes participant flow through the study.

Participants were recruited as a convenience sample through advertisements on social media and printed flyers at Goldsmiths University. The participants were entered into a lottery with the chance to win one of four £50 Amazon vouchers offered as an incentive for their participation. They engaged in the study between May 10 and September 1, 2023.

### Procedure

This pre-post study included pre-, post- and weekly assessments after digital art-making tasks on the Mindful of Art platform (See Figure 1). The registration page on the Mindful of Art website provided the participant information sheet and GDPR guidelines, directing participants to an initial online survey.^2^ There, they submitted demographic data, consent, and a registration survey before being able to access the course.

The Mindful of Art platform enables users to create new artworks by selecting and editing fragments of professional paintings with basic image-editing tools. Designed to empower those without formal artistic skills, the platform makes art creation accessible, supporting the democratization of art (Cavanagh, 2008).

The 8-week course introduced tasks progressively, starting with a simple square creation to familiarize participants with the tools and reduce creative anxiety (Mueller et al., 2012). Even if participants willingly joined the study, lack of creative drive and mood-related frontal lobe changes could have caused creative blocks (Flaherty, 2005).

Weekly tasks progressed in difficulty, from basic geometric shapes to organic ones, using weekly themes to foster creativity:

Week 1, Acceptance: Main: Create a square; Optional: Create rectangles.

Week 2, Appreciation: Main: Create shapes with diagonal lines; Optional: Combine with squares.

Week 3, Attention: Main: Create patterns; Optional: Add luminance effects.

Week 4, Compassion: Main: Create a path with color and luminance; Optional: Create two paths.

Week 5, Attunement: Main: Create round and squared shapes; Optional: Create concentric effects.

Week 6, Confidence: Main: Create a symbol representing home; Optional: Create a still life.

Week 7, Kindness: Main: Create a landscape; Optional: Add flowers/butterflies.

Week 8, Self-compassion: Main: Create a character; Optional: Freestyle.

The first task, despite its simplicity, was challenging for many participants. By week eight, however, they demonstrated improved control over colors, shapes, and chiaroscuro, indicating significant progress in digital art skills.

After each task, participants completed a brief post-art intervention survey assessing flow, arousal, positivity, and anxiety before submitting their artwork.

Participants were encouraged (but not required) to complete tasks weekly. Given the study’s extended duration, we expected some drop-off, so we treated task completion as a moderator to account for data churn. The final survey was sent 8 weeks after registration, regardless of number of tasks completed.

### Registration survey—demographics, art interest, self-perceived levels of creativity and anxiety

In the pre-course survey, participants provided demographic details (age, gender, and ethnicity) and completed the Vienna Art Interest and Art Knowledge Questions (VAIAK; Specker et al., 2020) to assess their engagement in art. The VAIAK measures interest and knowledge of art, with scores above 50 indicating art expertise.

Self-perception of creativity was assessed using the Short Scale of Creative Self (SSCS; Zielińska et al., 2022). The SSCS has 11 questions on a 5-point Likert scale, from which 6 questions measure creative self-efficacy, and 5 questions measure creative personal identity. A score of around 33 indicates a moderate self-perception of creativity, while scores around 11 would suggest a low self-perception of creativity.

Anxiety levels were measured using the STAI Y-2 (Spielberger et al., 1971) to evaluate each participant’s baseline of anxiety at the start of the course. The STAI Y-2 consists of 20 questions, scored on a 4-point Likert scale (“almost never,” “sometimes,” “often,” “almost always”), with scores ranging from 20 to 80. Results divided participants into three anxiety groups: Low (20–37), Moderate (38–44), and High (45–80), based on the scale’s standard classification.

### Weekly post-task survey—flow, arousal, positivity and anxiety

Engagement was measured using the Dispositional Flow Scale-2 (DFS-2; Jackson and Eklund, 2002), which assesses perceived skill and control over the activity. The 36-item scale is scored on a 5-point Likert scale (“never” to “always”), with scores ranging from 36 to 180. Flow is theorized to occur when the challenge level matches or slightly exceeds the participant’s skill level.

The Affect Grid (Russell et al., 1989) assessed pleasure–displeasure and arousal–sleepiness on a 9 × 9 grid (scores 1–81), with respondents marking their current emotional state.

Anxiety was measured using the STAI Y-1 (Spielberger et al., 1971), a 20-item scale scored on a 4-point Likert scale (“not at all” to “very much so”), with scores ranging from 20 to 80.

### Final survey—anxiety and platform feedback

The final survey reassessed anxiety using the STAI Y-2 to compare pre- and post-course levels. Participants also rated their course satisfaction and likelihood of using the platform as a self-help tool, which is beyond the scope of this work.

### Data analysis

To test our first hypothesis, we used a moderation analysis (Tang et al., 2009) to examine whether anxiety reduction from the art intervention was influenced by moderator variables, including course completion, VAIAK, and SSCS scores. Ordinary least squares (OLS) regression assessed the impact of the intervention on anxiety reduction (STAI_change).

For the second hypothesis, we used a dose–response framework (McVay et al., 2019) to test if more interventions led to greater anxiety reduction. Pearson correlation analysis examined the relationship between weekly task completion and anxiety reduction.

For our third hypothesis, we used a longitudinal study framework (van Weel, 2005) to observe participants’ changes in their experience of flow and arousal as they engage in the digital art-making tasks over the duration of the study. We utilized Spearman’s correlation to analyze the weekly measurements due to the lack of a linear relationship between anxiety, arousal, positivity and flow.

Finally, for our fourth hypothesis we used a factorial study framework to compare the anxiety reduction effect between the low, moderate and high anxiety groups determined by the STAI Y-2 score obtained at the beginning of the study and after 8 weeks. We performed a Type III ANOVA to examine whether the intervention’s effect on anxiety varied by participants’ initial anxiety levels.

## Results

### Descriptive statistics

15 participants were excluded from the final sample because they only completed the initial survey. 5 participants did not submit their final survey but still had their weekly STAI score recorded, so we decided to keep them in. The final sample of 22 participants were on the lower spectrum of the VAIAK scale (range 19–55, M = 39.41, SD = 11.73). This indicates that, on average, they did not possess a high level of interest or knowledge in art, as their scores were lower than those reported for the lay group in the Specker et al. study (Specker et al., 2020).

However, the group scored high on the Short Scale of Creative Self (range 23–44, M = 35.09, SD = 5.62), suggesting a reasonable level of confidence in handling creative challenges.

At the start of the study, the participants’ STAI Y-2 scores divided them into three groups: 9 in the low-anxiety group, 4 in the moderate-anxiety group, and 9 in the high-anxiety group.

### Intervention effectiveness not correlated to participant’s art background

**Figure 2:**
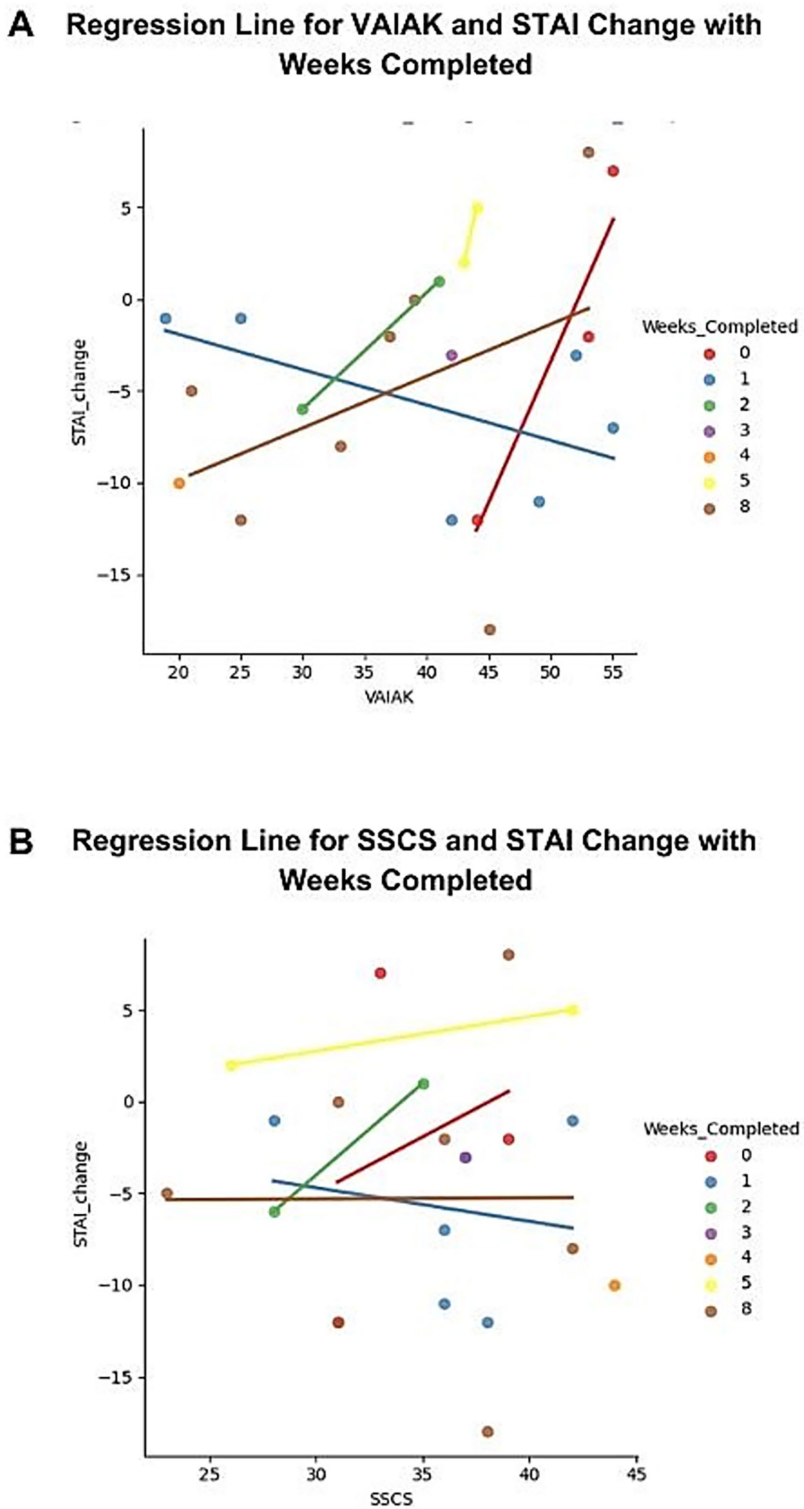
Influence of VAIAK and SSCS Scores on Anxiety Reduction. **(A)** Regression plot showing the moderation effect of VAIAK scores on anxiety reduction. **(B)** Regression plot showing the moderation effect of SSCS scores on anxiety reduction.

The figures created for this analysis include the number of weeks the intervention lasted, as the aim was to determine whether STAI Y-2 changes were influenced by VAIAK or SSCS scores. No statistically significant correlation was found between the effect of the art intervention on anxiety and the VAIAK or SSCS scores. Pearson correlation analyses showed that both VAIAK and SSCS were not related to STAI Y-2 changes (VAIAK, *r* = 0.22, *p* < 0.303; SSCS, *r* = −0.04, *p* < 0.846). This lack of correlation is evident in Figure 2.

### Digital art-making weekly task results

**Figure 3:**
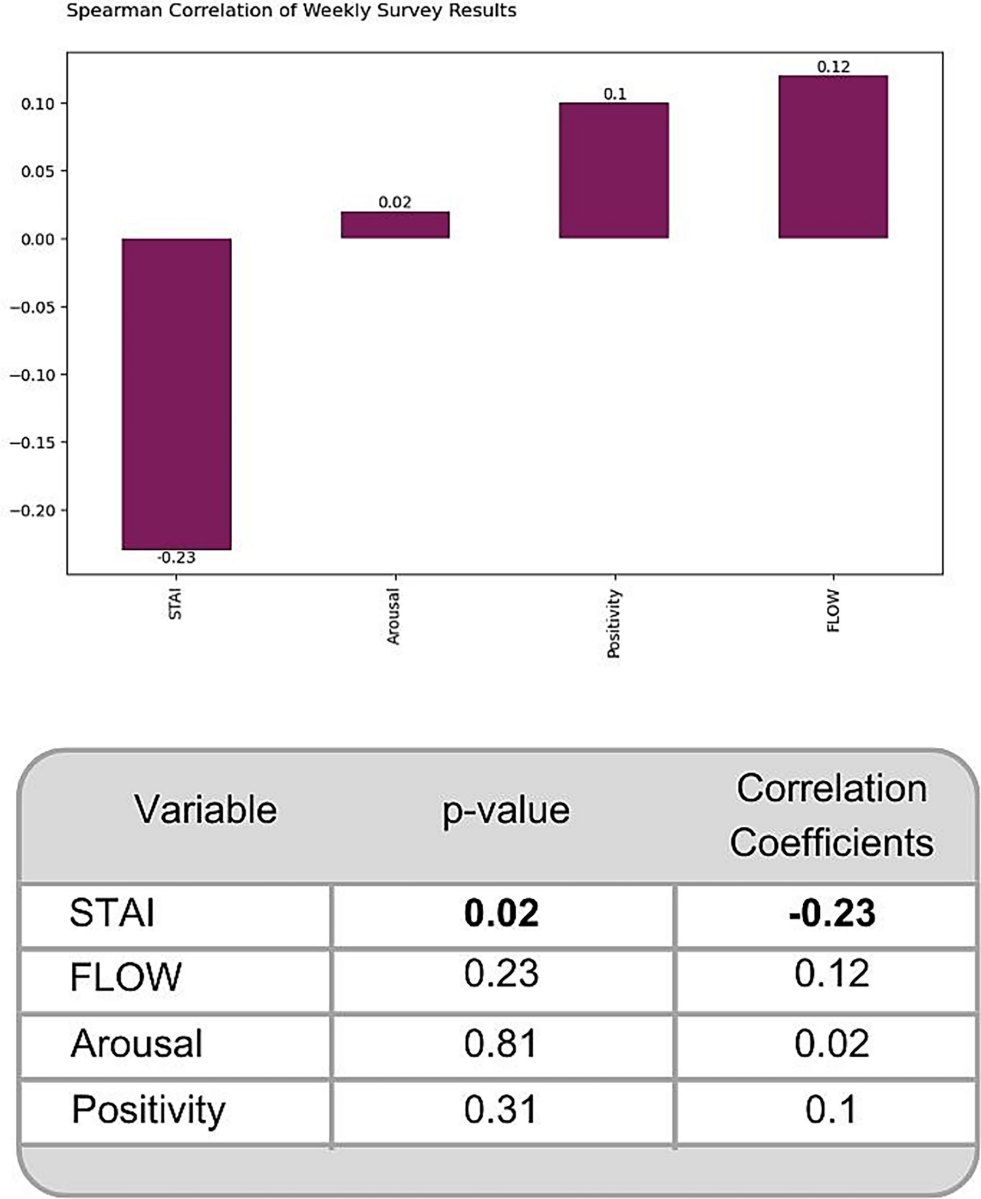
Spearman’s correlation showing the monotonic association of the weekly assessed variables: anxiety (STAI), arousal, positivity and flow.

Spearman’s correlation was used due to the non-linear relationship and differing variable scales. We expected a negative coefficient for STAI (indicating reduced anxiety) and positive coefficients for Arousal, Positivity, and Flow as the intervention progressed across weeks. While Figure 3 shows the expected direction of the trends, only the anxiety variable (STAI) showed a notable significant correlation (*r* − 0.23, *p*-value 0.02); other variables lacked strong or significant correlations across weeks. As this is an exploratory correlation analysis, no correction for multiple comparisons was applied to avoid overlooking potential relationships (Rothman, 1990; Saville, 1990).

### Art intervention for anxiety reduction

**Figure 4:**
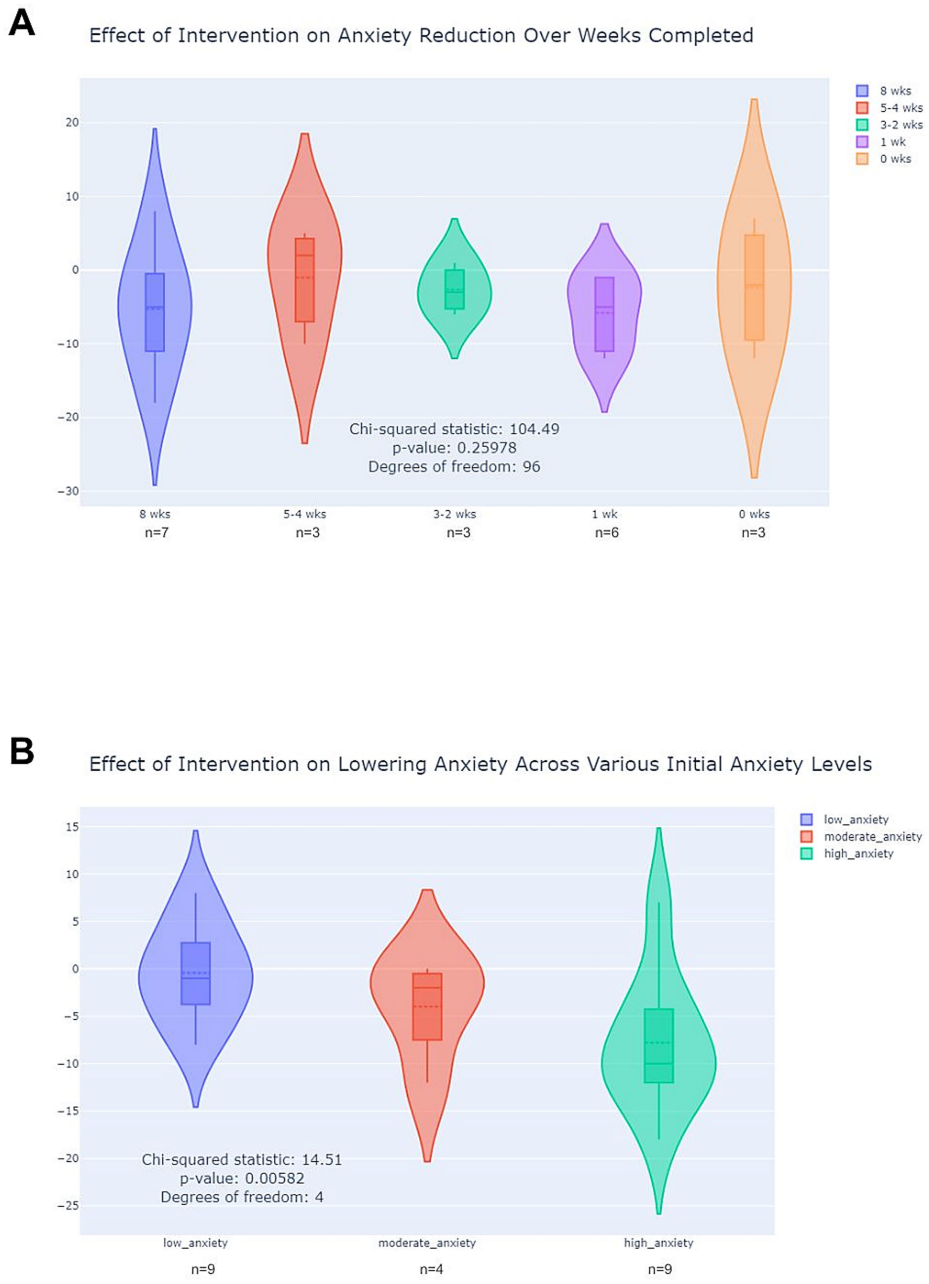
Effect of Intervention on Anxiety Over Time. **(A)** Violin plots showing anxiety changes by the number of weekly tasks completed. **(B)** Violin plots showing anxiety changes by initial anxiety levels at registration.

Our results indicated a very weak non-significant negative linear relationship (Figure 4A) between the number of weeks doing the digital art interventions and the decrease in anxiety levels (*𝑟* = − 0.03, 𝑝 < 0.882). This result was not in line with our initial hypothesis of seeing a strong relationship between anxiety reduction and frequency of digital art-making activities. Given that a linear relationship may not fully capture the nature of this association, we also conducted a chi-squared test to examine whether categorical differences in weeks completed were related to changes in anxiety levels. The test produced a chi-squared statistic of 𝜒^2^ = 104.49 with 𝑑𝑓 = 96 and a *p*-value of 𝑝 = 0.2598, indicating no statistically significant association. These findings suggest that, within our sample, the number of weeks participating in the intervention was not meaningfully associated with changes in anxiety. Given that 40% of the cohort already fell within the lower anxiety range at baseline, the lack of a significant association may reflect a limited scope for measurable improvement rather than an absence of intervention effect.

When we grouped participants by anxiety level, we observed that the anxiety reduction effect of the digital art interventions was significantly stronger within participants who reported higher levels of anxiety at the beginning of the study, as seen in Figure 4B. We conducted a Type III ANOVA to analyze the effects of individual differences (represented by participant ID) and the number of weeks doing the digital art interventions. The results showed that the number of weeks had a statistically significant effect on anxiety (𝑝 = 0.007548276). Additionally, the participant ID variable had an even smaller p-value (𝑝 = 5.811043e-08), indicating significant differences in anxiety levels between individuals. To further explore the relationship between pre- and post-intervention scores, we performed a Pearson correlation analysis, which revealed a strong positive relationship between pre- and post-intervention scores (𝑟 = 0.767, 𝑝 = 0.0001). This suggests that participants’ anxiety levels after the intervention were closely related to their initial levels. Additionally, we conducted a chi-squared test to examine if categorical differences in pre-intervention anxiety levels influenced post-intervention outcomes. The chi-squared test produced a statistic of 𝜒^2^ = 14.51 with 𝑑𝑓 = 4 and a 𝑝-value of 0.0058, indicating a statistically significant association. These findings suggest that initial anxiety levels significantly impacted post-intervention outcomes, reinforcing the notion that participants’ baseline state influenced their response to the digital art intervention.

## Discussion

This study explored whether regular digital art-making could improve mood and reduce anxiety. Trupp et al. (2022) found that even a brief (2-min) online art viewing reduced sadness, anxiety, and agitation during the first COVID-19 lockdown, though it did not increase positive feelings like happiness. Similarly, our 8-week intervention was associated with a reduction in anxiety levels but showed no strong correlation with positivity scores.

These findings suggest digital art-making could help reduce anxiety. While not statistically significant, they align with growing evidence of the arts’ positive impact on health and wellbeing, with art increasingly seen as a health promoter and alternative treatment (Fancourt and Finn, 2019).

Anxiety disorders are a major mental health burden (Wanjau et al., 2023), but many sufferers cannot afford therapy. Self-help treatments offer a more affordable alternative and have proven effective for anxiety (Haug et al., 2012). Our findings support the potential of self-help interventions to reduce anxiety.

Although intervention frequency can influence art therapy efficacy (Zhang et al., 2024), we found no significant link between anxiety reduction and digital art-making frequency. Digital art-making offers advantages over traditional methods, such as easier access and lower skill requirements, making it a promising self-help tool for anxiety and low mood. While traditional art therapy is well-established, some art therapists remain resistant to digital methods (Gussak and Nyce, 1999; Zubala et al., 2021). We encourage them to consider digital options to expand their reach.

Our findings align with previous studies showing that art interventions can serve as effective short-term support for improving mental wellbeing (Bu et al., 2022; Bone et al., 2024), as the number of art interventions was not a predictor of anxiety reduction. Furthermore, considering that even brief online art viewing can significantly reduce negative mood and anxiety (Trupp et al., 2023), it can be hypothesized that short digital art-making studies may also aid in stress and anxiety management. Our results tentatively suggest a potential anxiety reduction effect following a digital art-making course, with benefits scaling up with levels of anxiety, and we think that further research in this topic is certainly warranted.

It is advisable that future studies not only attempt replication but also implement more rigorous and controlled methodologies, including a pre-intervention survey to measure anxiety pre-task levels to assess the effectiveness of the art intervention. We measured trait anxiety and state anxiety using STAI Y-1 and STAI Y-2, but there are other anxiety assessment tools that could be used, like GAD-7, a validated screener for generalized anxiety symptoms (Spitzer et al., 2006) or VAS-A, a real-time subjective measure of anxiety intensity (Abend et al., 2014).

Replication studies should avoid an 8-week duration, as participant engagement declined after week 4. Future research studies should aim to recruit a larger and more diverse sample, incorporating a control group. Preferably, it should be a traditional art-making control group to allow for direct comparison with the digital art-making intervention.

Additionally, several participants reported they would have been more likely to continue if the course had been delivered through a mobile app. Future studies may therefore benefit from exploring app-based platforms to enhance accessibility and sustained engagement.

### Limitations of this study

This feasibility study aims to inspire further research on digital art-making. However, recruitment limitations resulted in a small sample of 22 participants, making findings non-generalizable. Another limitation was measuring anxiety only after each weekly task, missing immediate pre-task levels and providing an incomplete picture of the intervention’s effectiveness. A pre-intervention survey would have addressed this gap.

Conducted entirely remotely, this study falls under the category of an uncontrolled before-and-after study (Portela et al., 2015), suitable for a pilot but unable to detect confounders affecting post-intervention results. Regression to the mean is also a concern, as intervention effects may be transient, as observed in similar pre-post research (Marsden and Torgerson, 2012). Technical issues during registration and the first study week further reduced participant eligibility.

Another reason that might have deterred people from registering was the study’s 8-week length. There is evidence that a 4-week abbreviated mindfulness-based intervention (MBI) can have similar effects sizes as a standard 8-week MBI (Demarzo et al., 2017). Based on these results, there could be a possibility that shorter digital art-making interventions could keep their efficacy, making them more accessible.

We also failed to record changes in VAIAK and SSCS scores at the end of the 8-week course, which would have shown if the intervention impacted participants’ art appreciation and creativity.

Finally, we received feedback from participants indicating that they would have continued with digital art-making if the website had been an app. Unfortunately, the application we used at the time was only available through internet browsers.

## Conclusion

This feasibility study explored the potential of an 8-week digital art-making course as a self-help intervention for anxiety. While overall results were not statistically significant across the full sample, the intervention aligns with existing literature suggesting that even brief creative engagement can help reduce anxiety. The program showed promise as a low cost, accessible option for individuals seeking non-traditional mental health support, especially those who cannot access formal therapy.

Importantly, when participants were grouped by baseline anxiety levels, we found that the anxiety reducing effect of digital art-making was significantly stronger among individuals with higher initial anxiety. This subgroup demonstrated a statistically significant reduction in anxiety (𝑝 = 0.0075), suggesting that digital art interventions may be especially beneficial for those experiencing more severe anxiety symptoms.

In summary, this feasibility study tentatively suggests that digital art-making interventions may effectively reduce anxiety levels, complementing existing literature on the arts’ positive impact on mental health. Although our findings did not achieve statistical significance, they indicate a potential benefit worth exploring further. The limitations of a small sample size and lack of baseline measurements highlight the need for more rigorous research. Future studies with larger samples and stronger methodologies are essential to validate these preliminary findings.

Although no clear relationship was found between the frequency of art-making and anxiety reduction across the whole group, the results tentatively support the potential mental health benefits of accessible creative practices. These findings add to the growing body of evidence on the role of the arts in promoting mental wellbeing and support the use of digital platforms as scalable tools for intervention delivery.

That said, several limitations must be acknowledged. The small sample size, lack of a control group, and absence of pre-task anxiety measures limit the strength and generalizability of the findings. Additionally, participant engagement declined after week four, and feedback highlighted a preference for app-based delivery over web platforms, a consideration for future program design.

Future studies should aim for larger, more diverse samples and implement controlled designs comparing digital and traditional art-making formats. Incorporating baseline anxiety assessments and shorter intervention durations may improve engagement and data quality. Despite these constraints, the present study offers valuable preliminary insights and provides a strong rationale for further investigation into the role of digital art-making in supporting mental health. However, we acknowledge that individuals with low digital literacy may face barriers in accessing and fully engaging with this type of intervention, which should be considered in future program design.

## Data Availability

The data analysis conducted in Python and used in this study are included in the Supplementary material. Remaining data that supports the conclusions of this article will made made available by the authors, upon reasonable request.

## Footnotes

^1^ www.mfoart.org

^2^ www.qualtrics.com
