# Supplementary material for: The impact of digital art-making on anxiety: a feasibility study
Source: Front Psychol. 2025 Sep 15;16:1620583. doi: 10.3389/fpsyg.2025.1620583 (PMC12477173; doi:10.3389/fpsyg.2025.1620583)
Supplement: Supplementary file 1 [file Data_Sheet_1.pdf]

In [1]:
```
import seaborn as sns
import pandas as pd
%matplotlib inline
import matplotlib.pyplot as plt
import numpy as np
```

In [2]:
```
%%capture
pip install pandas scipy
```

In [3]:
```
%%capture
pip install -U kaleido
```

In [4]:
```
%%capture
pip install --upgrade seaborn
```

In [5]:
```
import seaborn as sns
print(sns.__version__)
```

```
0.13.2
```

# Initial Data Validation

The data for this analysis has been sourced from three distinct CSV files. Each file contains relevant information, which has been restructured and integrated. The configuration process involved aligning data formats, ensuring consistency across datasets, and combining key variables.

## MFoA data

This first dataset contains all data collected at the beginning and end of the study using the Qualtrics platform.

In [10]:
```
url = 'https://raw.githubusercontent.com/lauricocha/Digital-Art-Making-Research-2024/main/MFoA-data.csv'
MFOA = pd.read_csv(url)
```

In [11]:
```
MFOA.info()
```

```
<class 'pandas.core.frame.DataFrame'>
RangeIndex: 22 entries, 0 to 21
Data columns (total 16 columns):
 #   Column                 Non-Null Count  Dtype  
---  ------                 --------------  -----  
 0   ID                     22 non-null     object 
 1   Age                    22 non-null     int64  
 2   Gender                 22 non-null     object 
 3   Ethnicity ID           22 non-null     object 
 4   VAIAK                  22 non-null     int64  
 5   SSCS                   22 non-null     int64  
 6   Initial_Anxiety_level  22 non-null     object 
 7   STAI-T0                22 non-null     int64  
 8   STAI-T1                17 non-null     float64
 9   Final_Anxiety_Level    22 non-null     object 
 10  STAI-change            22 non-null     int64  
 11  Last_Recorded_STAI     22 non-null     int64  
 12  Weeks_done             22 non-null     object 
 13  Weeks_Completed        22 non-null     int64  
 14  Liking                 17 non-null     float64
 15  Application            17 non-null     float64
dtypes: float64(3), int64(7), object(6)
memory usage: 2.9+ KB
```

In [12]:
```
MFOA.head(23)
```

Out[12]:

| | ID | Age | Gender | Ethnicity ID | VAIAK | SSCS | Initial_Anxiety_level | STAI-T0 | STAI-T1 | Final_Anxiety_Level | STAI-change | Last_Recorded_STAI | Weeks_do |
|---|---|---|---|---|---|---|---|---|---|---|---|---|---|
| **0** | AMF1970039 | 52 | Female | HispLat | 53 | 39 | low_anxiety | 29 | 37.0 | low_anxiety_(20-37) | 8 | 37 | 8 w |
| **1** | BNF2002007 | 20 | Female | Asian | 37 | 36 | low_anxiety | 25 | 23.0 | low_anxiety_(20-37) | -2 | 23 | 8 w |
| **2** | NKM1972043 | 50 | Male | Mixed | 19 | 28 | moderate_anxiety | 41 | NaN | moderate_anxiety_(38-44) | -1 | 40 | 1 |
| **3** | CDF1998025 | 24 | Female | White | 21 | 23 | high_anxiety | 52 | 47.0 | high_anxiety_(45-80) | -5 | 47 | 8 w |
| **4** | CEM1994045 | 28 | Male | White | 25 | 31 | high_anxiety | 72 | 60.0 | high_anxiety_(45-80) | -12 | 60 | 8 w |
| **5** | EVM1965040 | 57 | Male | White | 45 | 38 | high_anxiety | 52 | 34.0 | low_anxiety_(20-37) | -18 | 34 | 8 w |
| **6** | LVF1995046 | 28 | Female | Mixed | 39 | 31 | moderate_anxiety | 40 | 40.0 | moderate_anxiety_(38-44) | 0 | 40 | 8 w |
| **7** | VVR2003016 | 19 | Prefer not to say | HispLat | 33 | 42 | low_anxiety | 34 | 26.0 | low_anxiety_(20-37) | -8 | 26 | 8 w |
| **8** | HKF1990010 | 32 | Female | Asian | 44 | 42 | low_anxiety | 27 | 32.0 | low_anxiety_(20-37) | 5 | 32 | 5-4 w |
| **9** | YHF1991019 | 31 | Female | Asian | 43 | 26 | low_anxiety | 32 | NaN | low_anxiety_(20-37) | 2 | 34 | 5-4 w |
| **10** | SVM1997033 | 25 | Male | HispLat | 20 | 44 | high_anxiety | 56 | 46.0 | high_anxiety_(45-80) | -10 | 46 | 5-4 w |
| **11** | SHF1961006 | 61 | Female | Other | 42 | 37 | low_anxiety | 29 | 26.0 | low_anxiety_(20-37) | -3 | 26 | 3-2 w |
| **12** | CCF1950049 | 72 | Female | White | 41 | 35 | low_anxiety | 24 | 25.0 | low_anxiety_(20-37) | 1 | 25 | 3-2 w |
| **13** | ROF1972041 | 51 | Female | White | 30 | 28 | low_anxiety | 31 | 25.0 | low_anxiety_(20-37) | -6 | 25 | 3-2 w |
| **14** | JMM1966029 | 56 | Male | HispLat | 25 | 42 | low_anxiety | 21 | NaN | low_anxiety_(20-37) | -1 | 20 | 1 |
| **15** | BFO1999021 | 23 | Non-binary | White | 55 | 36 | high_anxiety | 48 | NaN | moderate_anxiety_(38-44) | -7 | 41 | 1 |
| **16** | LMF1997018 | 26 | Female | HispLat | 49 | 36 | high_anxiety | 52 | NaN | moderate_anxiety_(38-44) | -11 | 41 | 1 |
| **17** | akF1992022 | 31 | Female | White | 44 | 31 | high_anxiety | 60 | 48.0 | high_anxiety_(45-80) | -12 | 48 | 0 w |
| **18** | GCM1969037 | 53 | Male | Asian | 42 | 38 | moderate_anxiety | 42 | 30.0 | low_anxiety_(20-37) | -12 | 30 | 1 |
| **19** | RHM1998036 | 24 | Male | White | 53 | 39 | high_anxiety | 53 | 51.0 | high_anxiety_(45-80) | -2 | 51 | 0 w |
| **20** | TBF1998044 | 25 | Female | White | 52 | 37 | moderate_anxiety | 39 | 36.0 | low_anxiety_(20-37) | -3 | 36 | 1 |
| **21** | JSF1999053 | 23 | Female | White | 55 | 33 | high_anxiety | 48 | 55.0 | high_anxiety_(45-80) | 7 | 55 | 0 w |

In [13]:
```
MFOA.describe()
```

Out[13]:

| | Age | VAIAK | SSCS | STAI-T0 | STAI-T1 | STAI-change | Last_Recorded_STAI | Weeks_Completed | Liking | Application |
|---|---|---|---|---|---|---|---|---|---|---|
| **count** | 22.000000 | 22.000000 | 22.000000 | 22.000000 | 17.000000 | 22.000000 | 22.000000 | 22.000000 | 17.000000 | 17.000000 |
| **mean** | 36.863636 | 39.409091 | 35.090909 | 41.227273 | 37.705882 | -4.090909 | 37.136364 | 3.772727 | 7.705882 | 7.058824 |
| **std** | 16.063254 | 11.733790 | 5.622312 | 13.479903 | 11.623063 | 6.739430 | 10.929625 | 3.264992 | 1.992633 | 2.771971 |
| **min** | 19.000000 | 19.000000 | 23.000000 | 21.000000 | 23.000000 | -18.000000 | 20.000000 | 0.000000 | 4.000000 | 2.000000 |
| **25%** | 24.250000 | 30.750000 | 31.000000 | 29.500000 | 26.000000 | -9.500000 | 27.000000 | 1.000000 | 7.000000 | 4.000000 |
| **50%** | 29.500000 | 42.000000 | 36.000000 | 40.500000 | 36.000000 | -3.000000 | 36.500000 | 2.500000 | 8.000000 | 7.000000 |
| **75%** | 51.750000 | 48.000000 | 38.750000 | 52.000000 | 47.000000 | -0.250000 | 44.750000 | 8.000000 | 9.000000 | 10.000000 |
| **max** | 72.000000 | 55.000000 | 44.000000 | 72.000000 | 60.000000 | 8.000000 | 60.000000 | 8.000000 | 10.000000 | 10.000000 |

In [14]:
```
MFOA.isna()
```

Out[14]:

| | ID | Age | Gender | Ethnicity ID | VAIAK | SSCS | Initial_Anxiety_level | STAI-T0 | STAI-T1 | Final_Anxiety_Level | STAI-change | Last_Recorded_STAI | Weeks_done | Weel |
|---|---|---|---|---|---|---|---|---|---|---|---|---|---|---|
| **0** | False | False | False | False | False | False | False | False | False | False | False | False | False | |
| **1** | False | False | False | False | False | False | False | False | False | False | False | False | False | |
| **2** | False | False | False | False | False | False | False | False | True | False | False | False | False | |
| **3** | False | False | False | False | False | False | False | False | False | False | False | False | False | |
| **4** | False | False | False | False | False | False | False | False | False | False | False | False | False | |
| **5** | False | False | False | False | False | False | False | False | False | False | False | False | False | |
| **6** | False | False | False | False | False | False | False | False | False | False | False | False | False | |
| **7** | False | False | False | False | False | False | False | False | False | False | False | False | False | |
| **8** | False | False | False | False | False | False | False | False | False | False | False | False | False | |
| **9** | False | False | False | False | False | False | False | False | True | False | False | False | False | |
| **10** | False | False | False | False | False | False | False | False | False | False | False | False | False | |
| **11** | False | False | False | False | False | False | False | False | False | False | False | False | False | |
| **12** | False | False | False | False | False | False | False | False | False | False | False | False | False | |
| **13** | False | False | False | False | False | False | False | False | False | False | False | False | False | |
| **14** | False | False | False | False | False | False | False | False | True | False | False | False | False | |
| **15** | False | False | False | False | False | False | False | False | True | False | False | False | False | |
| **16** | False | False | False | False | False | False | False | False | True | False | False | False | False | |
| **17** | False | False | False | False | False | False | False | False | False | False | False | False | False | |
| **18** | False | False | False | False | False | False | False | False | False | False | False | False | False | |
| **19** | False | False | False | False | False | False | False | False | False | False | False | False | False | |
| **20** | False | False | False | False | False | False | False | False | False | False | False | False | False | |
| **21** | False | False | False | False | False | False | False | False | False | False | False | False | False | |

In [15]:
```python
MFOA.info()
```

```
<class 'pandas.core.frame.DataFrame'>
RangeIndex: 22 entries, 0 to 21
Data columns (total 16 columns):
 #   Column                 Non-Null Count  Dtype  
---  ------                 --------------  -----  
 0   ID                     22 non-null     object 
 1   Age                    22 non-null     int64  
 2   Gender                 22 non-null     object 
 3   Ethnicity ID           22 non-null     object 
 4   VAIAK                  22 non-null     int64  
 5   SSCS                   22 non-null     int64  
 6   Initial_Anxiety_level  22 non-null     object 
 7   STAI-T0                22 non-null     int64  
 8   STAI-T1                17 non-null     float64
 9   Final_Anxiety_Level    22 non-null     object 
 10  STAI-change            22 non-null     int64  
 11  Last_Recorded_STAI     22 non-null     int64  
 12  Weeks_done             22 non-null     object 
 13  Weeks_Completed        22 non-null     int64  
 14  Liking                 17 non-null     float64
 15  Application            17 non-null     float64
dtypes: float64(3), int64(7), object(6)
memory usage: 2.9+ KB
```

# MFoA data weekly

The second dataset includes all weekly self-assessment scores on STAI, Flow, Arousal, and Positivity for each participant.

In [18]:
```python
url2 = 'https://raw.githubusercontent.com/lauricocha/Digital-Art-Making-Research-2024/main/MFoA-data-weekly.csv'
Weekly = pd.read_csv(url2)
```

In [19]:
```python
Weekly.info()
```

```
<class 'pandas.core.frame.DataFrame'>
RangeIndex: 110 entries, 0 to 109
Data columns (total 6 columns):
 #   Column       Non-Null Count  Dtype  
---  ------       --------------  -----  
 0   Week_number  110 non-null    float64
 1   ID           110 non-null    object 
 2   STAI         110 non-null    int64  
 3   FLOW         110 non-null    int64  
 4   Arousal      110 non-null    int64  
 5   Positivity   110 non-null    int64  
dtypes: float64(1), int64(4), object(1)
memory usage: 5.3+ KB
```

In [20]:
```
Weekly.head(23)
```

Out[20]:

| | Week_number | ID | STAI | FLOW | Arousal | Positivity |
|---|---|---|---|---|---|---|
| **0** | 0.0 | AMF1970039 | 29 | 0 | 0 | 0 |
| **1** | 1.0 | AMF1970039 | 28 | 44 | 5 | 9 |
| **2** | 2.0 | AMF1970039 | 27 | 42 | 7 | 9 |
| **3** | 3.0 | AMF1970039 | 33 | 40 | 3 | 9 |
| **4** | 4.0 | AMF1970039 | 26 | 43 | 3 | 9 |
| **5** | 5.0 | AMF1970039 | 24 | 44 | 4 | 9 |
| **6** | 6.0 | AMF1970039 | 26 | 45 | 3 | 9 |
| **7** | 7.0 | AMF1970039 | 34 | 32 | 4 | 7 |
| **8** | 8.0 | AMF1970039 | 26 | 39 | 5 | 9 |
| **9** | 8.1 | AMF1970039 | 37 | 0 | 0 | 0 |
| **10** | 0.0 | BNF2002007 | 25 | 0 | 0 | 0 |
| **11** | 1.0 | BNF2002007 | 23 | 33 | 4 | 7 |
| **12** | 2.0 | BNF2002007 | 41 | 35 | 3 | 2 |
| **13** | 3.0 | BNF2002007 | 23 | 34 | 3 | 9 |
| **14** | 4.0 | BNF2002007 | 24 | 34 | 5 | 8 |
| **15** | 5.0 | BNF2002007 | 20 | 38 | 2 | 8 |
| **16** | 6.0 | BNF2002007 | 20 | 40 | 3 | 9 |
| **17** | 7.0 | BNF2002007 | 24 | 37 | 4 | 6 |
| **18** | 8.0 | BNF2002007 | 20 | 36 | 4 | 9 |
| **19** | 8.1 | BNF2002007 | 23 | 0 | 0 | 0 |
| **20** | 0.0 | CDF1998025 | 52 | 0 | 0 | 0 |
| **21** | 1.0 | CDF1998025 | 53 | 30 | 5 | 4 |
| **22** | 2.0 | CDF1998025 | 40 | 32 | 8 | 7 |

In [21]:
```
Weekly.describe()
```

Out[21]:

| | Week_number | STAI | FLOW | Arousal | Positivity |
|---|---|---|---|---|---|
| **count** | 110.000000 | 110.000000 | 110.000000 | 110.000000 | 110.000000 |
| **mean** | 3.683636 | 34.300000 | 25.818182 | 3.118182 | 4.818182 |
| **std** | 2.858611 | 10.532214 | 16.454654 | 2.478279 | 3.395036 |
| **min** | 0.000000 | 20.000000 | 0.000000 | 0.000000 | 0.000000 |
| **25%** | 1.000000 | 26.000000 | 0.000000 | 0.000000 | 0.000000 |
| **50%** | 3.000000 | 33.000000 | 33.000000 | 3.000000 | 6.000000 |
| **75%** | 6.000000 | 40.750000 | 38.000000 | 5.000000 | 7.000000 |
| **max** | 8.100000 | 72.000000 | 45.000000 | 9.000000 | 9.000000 |

In [22]:
```
Weekly.isna()
```

Out[22]:

| | Week_number | ID | STAI | FLOW | Arousal | Positivity |
|---|---|---|---|---|---|---|
| 0 | False | False | False | False | False | False |
| 1 | False | False | False | False | False | False |
| 2 | False | False | False | False | False | False |
| 3 | False | False | False | False | False | False |
| 4 | False | False | False | False | False | False |
| ... | ... | ... | ... | ... | ... | ... |
| 105 | False | False | False | False | False | False |
| 106 | False | False | False | False | False | False |
| 107 | False | False | False | False | False | False |
| 108 | False | False | False | False | False | False |
| 109 | False | False | False | False | False | False |

110 rows × 6 columns

## MFoA data Pre-Post Intervention

This third dataset shows the pre- and post-intervention data of participants who completed at least one digital art session.

In [25]:
```
url3 = 'https://raw.githubusercontent.com/lauricocha/Digital-Art-Making-Research-2024/main/MFoA-data-pre-post-intervention.csv'
WeeklyV2 = pd.read_csv(url3)
```

In [26]:
```
WeeklyV2.info()
```

```
<class 'pandas.core.frame.DataFrame'>
RangeIndex: 19 entries, 0 to 18
Data columns (total 5 columns):
 #   Column             Non-Null Count  Dtype
---  ------             --------------  -----
 0   ID                 19 non-null     object
 1   Initial_STAI       19 non-null     int64
 2   Pre_Intervention   19 non-null     int64
 3   Final_STAI         19 non-null     int64
 4   Post_Intervention  19 non-null     int64
dtypes: int64(4), object(1)
memory usage: 892.0+ bytes
```

In [27]:
```
WeeklyV2.head(22)
```

Out[27]:

| | ID | Initial_STAI | Pre_Intervention | Final_STAI | Post_Intervention |
|---|---|---|---|---|---|
| 0 | AMF1970039 | 29 | 1 | 37 | 1 |
| 1 | BNF2002007 | 25 | 1 | 23 | 1 |
| 2 | CDF1998025 | 52 | 3 | 47 | 3 |
| 3 | CEM1994045 | 72 | 3 | 60 | 3 |
| 4 | EVM1965040 | 52 | 3 | 34 | 1 |
| 5 | LVF1995046 | 40 | 2 | 40 | 2 |
| 6 | VVR2003016 | 34 | 1 | 26 | 1 |
| 7 | HKF1990010 | 27 | 1 | 32 | 1 |
| 8 | YHF1991019 | 32 | 1 | 34 | 1 |
| 9 | SVM1997033 | 56 | 3 | 46 | 3 |
| 10 | SHF1961006 | 29 | 1 | 26 | 1 |
| 11 | CCF1950049 | 24 | 1 | 25 | 1 |
| 12 | ROF1972041 | 31 | 1 | 25 | 1 |
| 13 | JMM1966029 | 21 | 1 | 20 | 1 |
| 14 | BFO1999021 | 48 | 3 | 41 | 2 |
| 15 | LMF1997018 | 52 | 3 | 41 | 2 |
| 16 | NKM1972043 | 41 | 2 | 40 | 2 |
| 17 | GCM1969037 | 42 | 2 | 30 | 1 |
| 18 | TBF1998044 | 39 | 2 | 36 | 1 |

In [28]:
```
WeeklyV2.describe()
```

Out[28]:

| | Initial_STAI | Pre_Intervention | Final_STAI | Post_Intervention |
|---|---|---|---|---|
| **count** | 19.000000 | 19.000000 | 19.000000 | 19.000000 |
| **mean** | 39.263158 | 1.842105 | 34.894737 | 1.526316 |
| **std** | 13.345170 | 0.898342 | 9.954869 | 0.772328 |
| **min** | 21.000000 | 1.000000 | 20.000000 | 1.000000 |
| **25%** | 29.000000 | 1.000000 | 26.000000 | 1.000000 |
| **50%** | 39.000000 | 2.000000 | 34.000000 | 1.000000 |
| **75%** | 50.000000 | 3.000000 | 40.500000 | 2.000000 |
| **max** | 72.000000 | 3.000000 | 60.000000 | 3.000000 |

In [29]:

```
WeeklyV2.isna()
```

Out[29]:

| | ID | Initial_STAI | Pre_Intervention | Final_STAI | Post_Intervention |
|---|---|---|---|---|---|
| **0** | False | False | False | False | False |
| **1** | False | False | False | False | False |
| **2** | False | False | False | False | False |
| **3** | False | False | False | False | False |
| **4** | False | False | False | False | False |
| **5** | False | False | False | False | False |
| **6** | False | False | False | False | False |
| **7** | False | False | False | False | False |
| **8** | False | False | False | False | False |
| **9** | False | False | False | False | False |
| **10** | False | False | False | False | False |
| **11** | False | False | False | False | False |
| **12** | False | False | False | False | False |
| **13** | False | False | False | False | False |
| **14** | False | False | False | False | False |
| **15** | False | False | False | False | False |
| **16** | False | False | False | False | False |
| **17** | False | False | False | False | False |
| **18** | False | False | False | False | False |

# Assessing VAIAK and SSCS influence on STAI trends

## Regression Lines for SSCS and VAIAK scores changes in STAI level before and after intervention

In [32]:

```
columns_to_drop = ['Age', 'STAI-T0', 'STAI-T1', 'Liking', 'Application', 'Gender', 'Ethnicity ID', 'Weeks_Completed', 'ID', 'Final_Anxiety_
Artsy = MFOA.drop(columns_to_drop, axis=1)

Artsy
```

Out[32]:

| | VAIAK | SSCS | STAI-change |
|---|---|---|---|
| **0** | 53 | 39 | 8 |
| **1** | 37 | 36 | -2 |
| **2** | 19 | 28 | -1 |
| **3** | 21 | 23 | -5 |
| **4** | 25 | 31 | -12 |
| **5** | 45 | 38 | -18 |
| **6** | 39 | 31 | 0 |
| **7** | 33 | 42 | -8 |
| **8** | 44 | 42 | 5 |
| **9** | 43 | 26 | 2 |
| **10** | 20 | 44 | -10 |
| **11** | 42 | 37 | -3 |
| **12** | 41 | 35 | 1 |
| **13** | 30 | 28 | -6 |
| **14** | 25 | 42 | -1 |
| **15** | 55 | 36 | -7 |
| **16** | 49 | 36 | -11 |
| **17** | 44 | 31 | -12 |
| **18** | 42 | 38 | -12 |
| **19** | 53 | 39 | -2 |
| **20** | 52 | 37 | -3 |
| **21** | 55 | 33 | 7 |

In [33]:

```
import pandas as pd
import statsmodels.formula.api as smf
import seaborn as sns
import matplotlib.pyplot as plt
import shutil

# Read the CSV file
url = 'https://raw.githubusercontent.com/lauricocha/Digital-Art-Making-Research-2024/main/MFoA-data.csv'
MFOA = pd.read_csv(url)

# Ensure 'Weeks_Completed' is treated as a categorical variable
MFOA['Weeks_Completed'] = MFOA['Weeks_Completed'].astype('category')

# Calculate 'STAI_change' as the difference between 'Last_Recorded_STAI' and 'STAI-T0'
MFOA['STAI_change'] = MFOA['Last_Recorded_STAI'] - MFOA['STAI-T0']

# Check for any missing values in critical columns and handle them if necessary
MFOA = MFOA.dropna(subset=['STAI_change', 'Weeks_Completed', 'VAIAK', 'SSCS'])

# Fit the model
formula = 'STAI_change ~ Weeks_Completed * VAIAK + Weeks_Completed * SSCS'
model = smf.ols(formula, data=MFOA).fit()

# Print the summary of the model
print(model.summary())

# Plotting the regression lines and saving them

# Regression line for VAIAK and STAI_change
sns.lmplot(x='VAIAK', y='STAI_change', hue='Weeks_Completed', data=MFOA, ci=None, palette='Set1')
plt.title('Regression Line for VAIAK and STAI_change with Weeks_Completed')
plt.savefig('regression_VAIAK_STAI_change.png')  # Save the plot as a .png file
plt.show()

# Regression line for SSCS and STAI_change
sns.lmplot(x='SSCS', y='STAI_change', hue='Weeks_Completed', data=MFOA, ci=None, palette='Set1')
plt.title('Regression Line for SSCS and STAI_change with Weeks_Completed')
plt.savefig('regression_SSCS_STAI_change.png')  # Save the plot as a .png file
plt.show()

# Code to display the download links for the saved images
from IPython.display import FileLink, display

# Display the download links
display(FileLink(r'regression_VAIAK_STAI_change.png'))
display(FileLink(r'regression_SSCS_STAI_change.png'))
```

```
                            OLS Regression Results
==============================================================================
Dep. Variable:            STAI_change   R-squared:                       0.579
Model:                            OLS   Adj. R-squared:                 -0.264
Method:                 Least Squares   F-statistic:                    0.6865
Date:                Sat, 22 Feb 2025   Prob (F-statistic):              0.740
Time:                        20:18:24   Log-Likelihood:                -63.175
No. Observations:                  22   AIC:                             156.3
Df Residuals:                       7   BIC:                             172.7
Df Model:                          14
Covariance Type:            nonrobust
================================================================================================
                                  coef    std err          t      P>|t|      [0.025      0.975]
------------------------------------------------------------------------------------------------
Intercept                     -67.9571     51.480     -1.320      0.228    -189.688      53.774
Weeks_Completed[T.1]           70.2786     58.136      1.209      0.266     -67.191     207.748
Weeks_Completed[T.2]            0.9294      1.117      0.832      0.433      -1.713       3.571
Weeks_Completed[T.3]            0.0057      0.005      1.188      0.274      -0.006       0.017
Weeks_Completed[T.4]            0.0251      0.019      1.304      0.233      -0.020       0.071
Weeks_Completed[T.5]           -0.0090      0.034     -0.264      0.799      -0.090       0.072
Weeks_Completed[T.8]           64.3750     54.159      1.189      0.273     -63.691     192.441
VAIAK                           1.8857      1.104      1.708      0.131      -0.725       4.496
Weeks_Completed[T.1]:VAIAK     -2.0781      1.129     -1.841      0.108      -4.747       0.591
Weeks_Completed[T.2]:VAIAK     -4.2448      5.475     -0.775      0.464     -17.192       8.702
Weeks_Completed[T.3]:VAIAK      0.2412      0.203      1.188      0.274      -0.239       0.721
Weeks_Completed[T.4]:VAIAK      0.5014      0.384      1.304      0.233      -0.408       1.410
Weeks_Completed[T.5]:VAIAK     -0.3125      1.302     -0.240      0.817      -3.390       2.765
Weeks_Completed[T.8]:VAIAK     -1.3700      1.167     -1.174      0.279      -4.130       1.390
SSCS                           -0.8714      1.554     -0.561      0.592      -4.545       2.803
Weeks_Completed[T.1]:SSCS       0.8605      1.734      0.496      0.635      -3.239       4.960
Weeks_Completed[T.2]:SSCS       5.5786      6.475      0.862      0.417      -9.731      20.888
Weeks_Completed[T.3]:SSCS       0.2125      0.179      1.188      0.274      -0.211       0.636
Weeks_Completed[T.4]:SSCS       1.1030      0.846      1.304      0.233      -0.897       3.103
Weeks_Completed[T.5]:SSCS       0.9606      1.738      0.553      0.598      -3.148       5.069
Weeks_Completed[T.8]:SSCS       0.2780      1.685      0.165      0.874      -3.707       4.263
==============================================================================
Omnibus:                       21.421   Durbin-Watson:                   1.627
Prob(Omnibus):                  0.000   Jarque-Bera (JB):               32.395
Skew:                          -1.768   Prob(JB):                     9.24e-08
Kurtosis:                       7.779   Cond. No.                     3.65e+17
==============================================================================

Notes:
[1] Standard Errors assume that the covariance matrix of the errors is correctly specified.
[2] The smallest eigenvalue is 5.73e-31. This might indicate that there are
strong multicollinearity problems or that the design matrix is singular.
```

Regression Line for VAIAK and STAI_change with Weeks_Completed

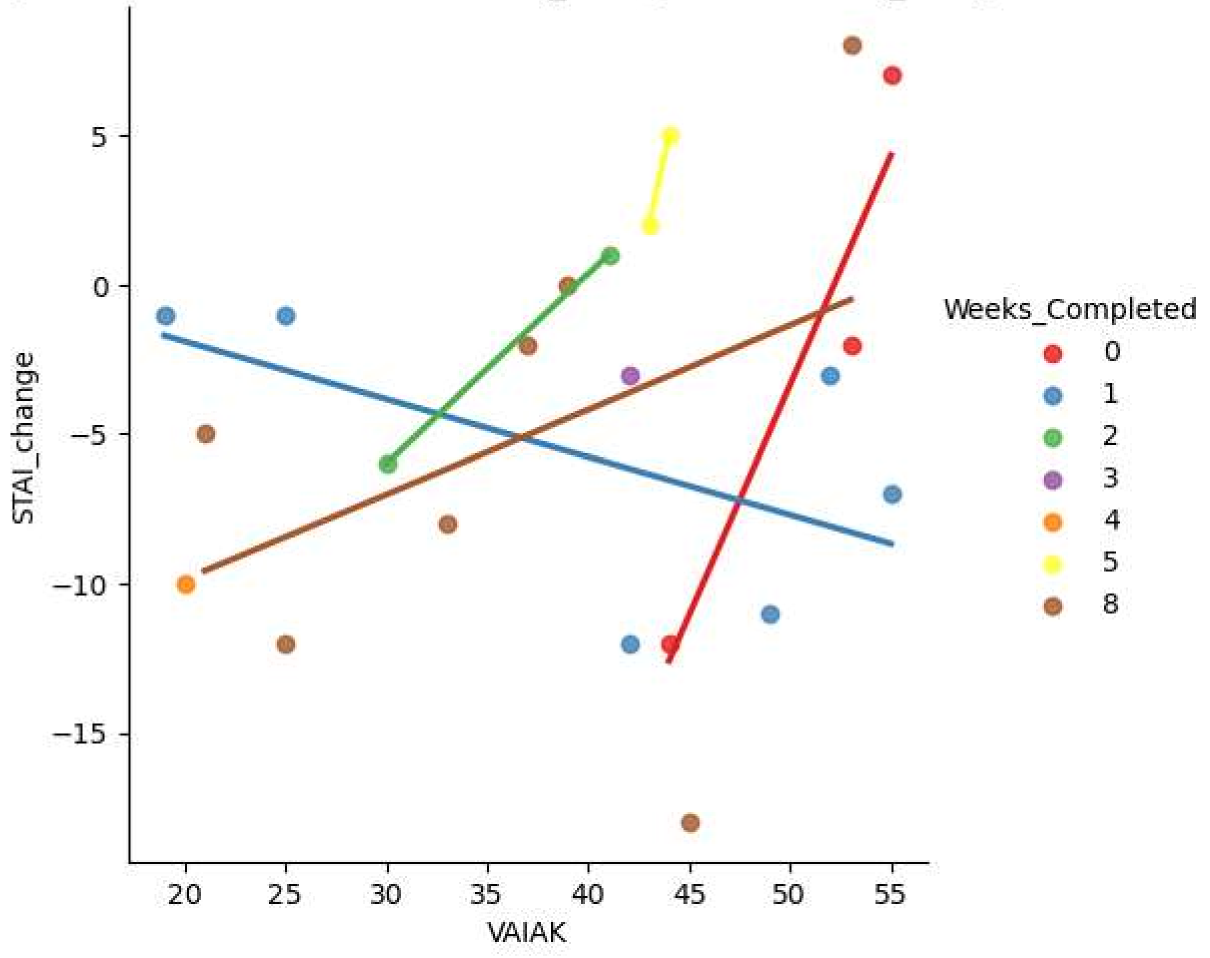

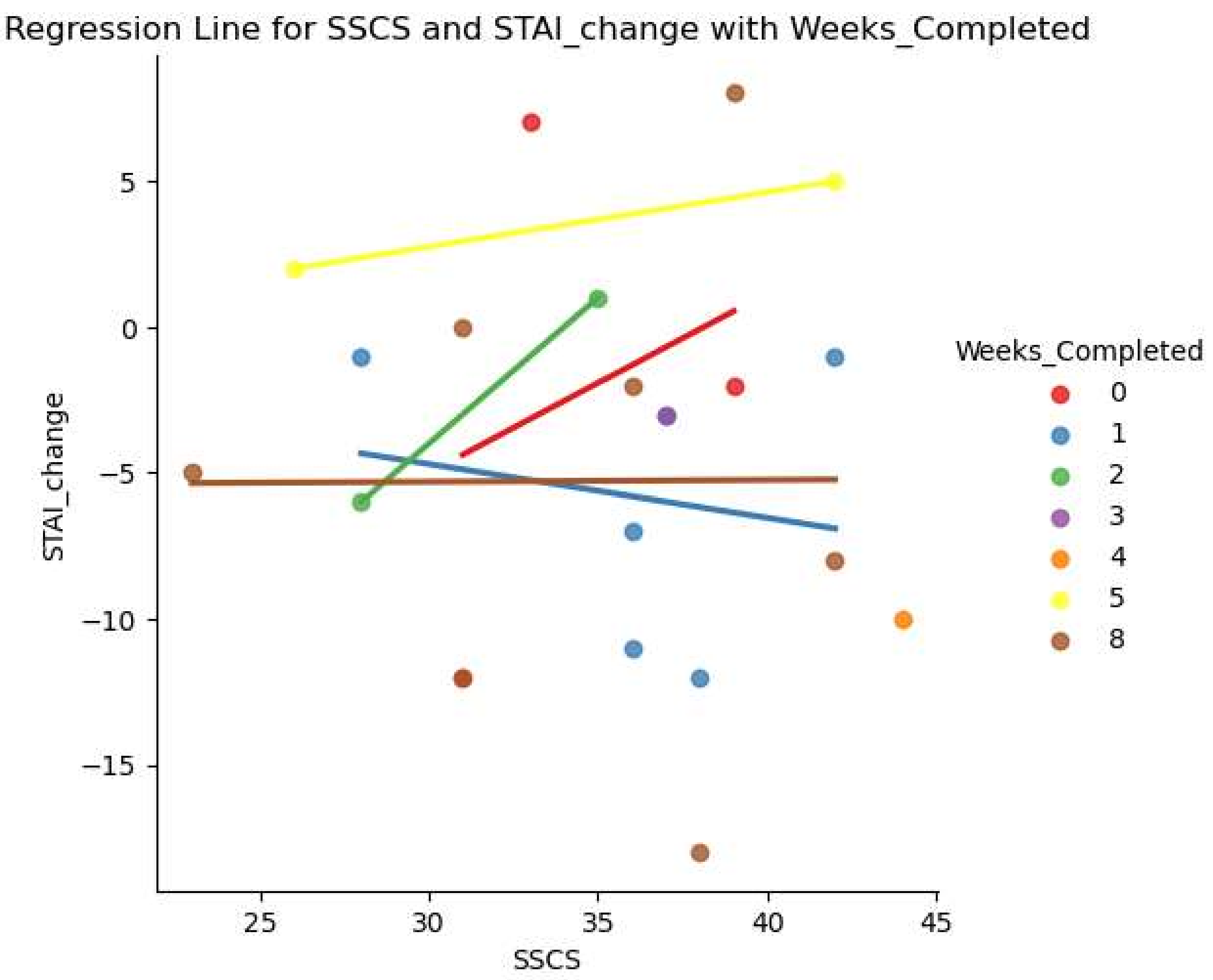


regression_VAIAK_STAI_change.png
regression_SSCS_STAI_change.png

## Comparison of VAIAK, SSCS, and STAI using Correlation Coefficients and P-values

In [35]:
```
Artsy.corr()
```

Out[35]:

| | VAIAK | SSCS | STAI-change |
|---|---|---|---|
| **VAIAK** | 1.000000 | 0.220286 | 0.229920 |
| **SSCS** | 0.220286 | 1.000000 | -0.043757 |
| **STAI-change** | 0.229920 | -0.043757 | 1.000000 |

## Heatmap of Correlation Coefficients: VAIAK, SSCS, and STAI

In [37]:
```
corr = Artsy.corr().round(2)

# plot the heatmap
plt.figure(figsize = (8,6))

#viz the heatmap
sns.heatmap(corr, annot= True)
```

Out[37]: <Axes: >

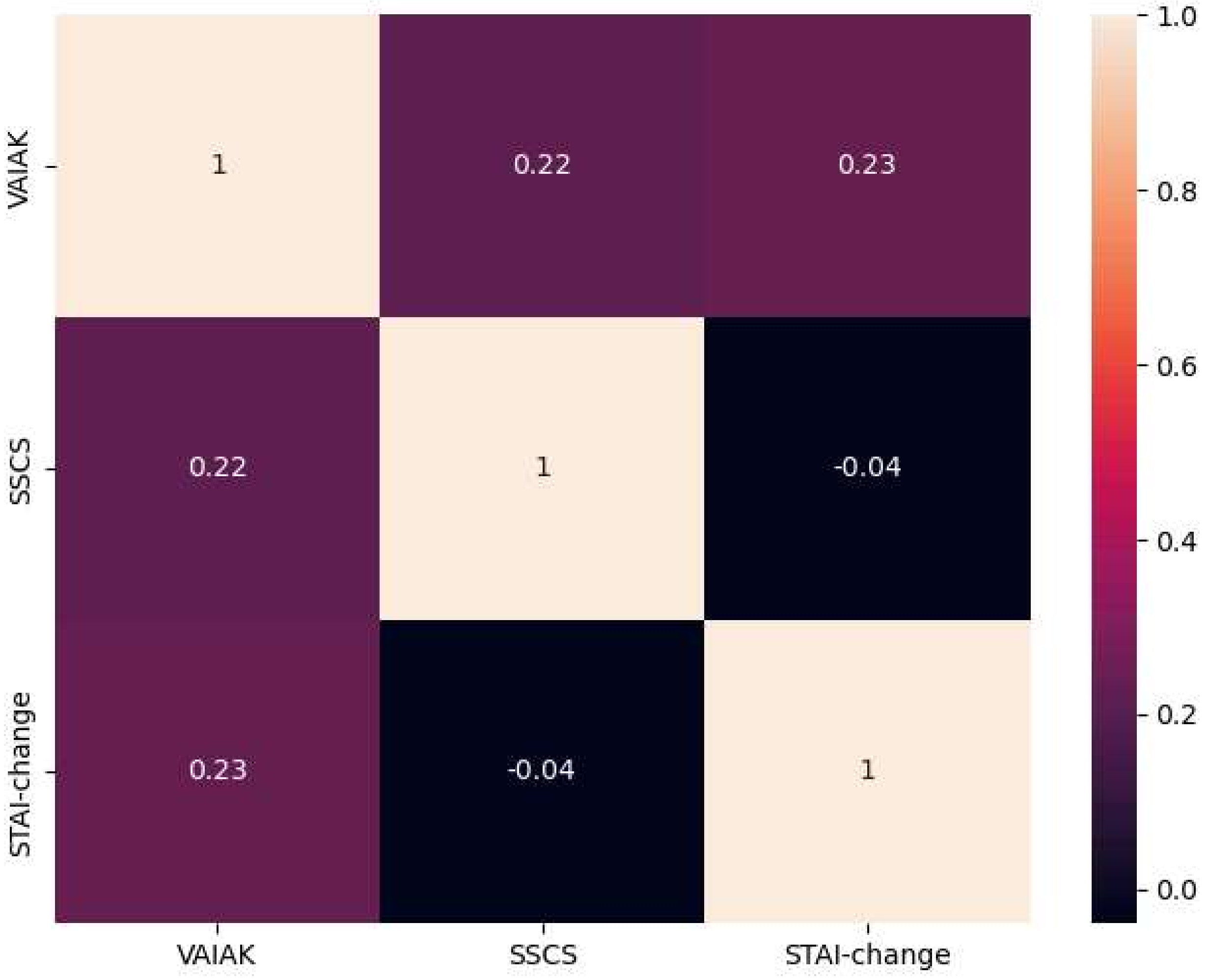


## P-Value Heatmap for Correlation Analysis: VAIAK, SSCS, and STAI

In [39]:
```
import numpy as np
import pandas as pd
import seaborn as sns
import scipy.stats as stats

# Calculate the correlation coefficients
correlation_matrix = Artsy.corr()

# Calculate the p-values
p_values = Artsy.apply(lambda x: Artsy.apply(lambda y: stats.pearsonr(x, y)[1]))

# Create a heatmap of the p-values
pvalue_heatmap = sns.heatmap(p_values, annot=True, cmap='rocket', fmt='.2f')
```

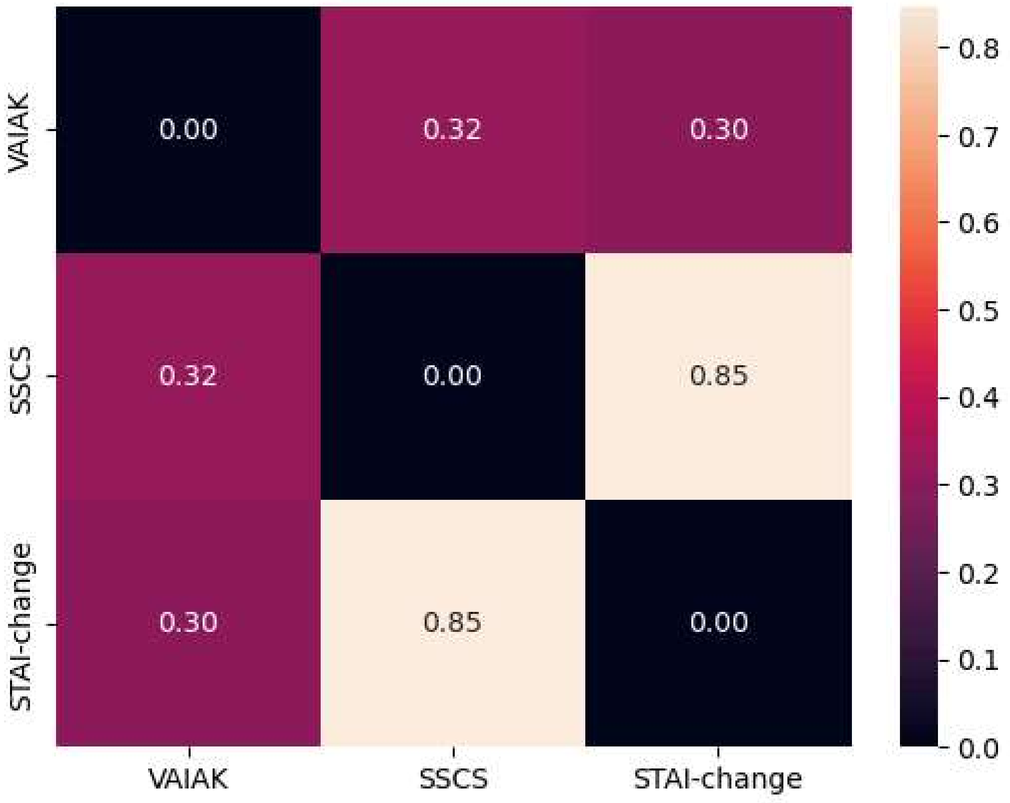


# Effect of intervention on anxiety reduction over weeks completed

In [204…
```
import plotly.graph_objects as go
import pandas as pd
import plotly.io as pio
from IPython.display import Image

# Read the CSV file
url = 'https://raw.githubusercontent.com/lauricocha/Digital-Art-Making-Research-2024/main/MFoA-data.csv'
```

```
df = pd.read_csv(url)

fig = go.Figure()

Weeks_done = ['8 wks', '5-4 wks', '3-2 wks', '1 wk', '0 wks']

for week in Weeks_done:
    fig.add_trace(go.Violin(x=df['Weeks_done'][df['Weeks_done'] == week],
                            y=df['STAI-change'][df['Weeks_done'] == week],
                            name=week,
                            box_visible=True,
                            meanline_visible=True))

fig.update_layout(
    title="Effect of Intervention on Anxiety Reduction<br>Over Weeks Completed",
    title_font=dict(size=22)
)

fig.add_annotation(
    text="Chi-squared statistic: 104.49<br>P-value: 0.25978<br>Degrees of freedom: 96",
    x=2,  # Adjust the x and y values for proper positioning
    y=-24,
    showarrow=False,
    font=dict(size=16)  # Adjust the font size as needed
)

# Save the figure as a static image using kaleido
pio.write_image(fig, "plotly_figure_violin.png")

# Display the static image
display(Image("plotly_figure_violin.png"))
```

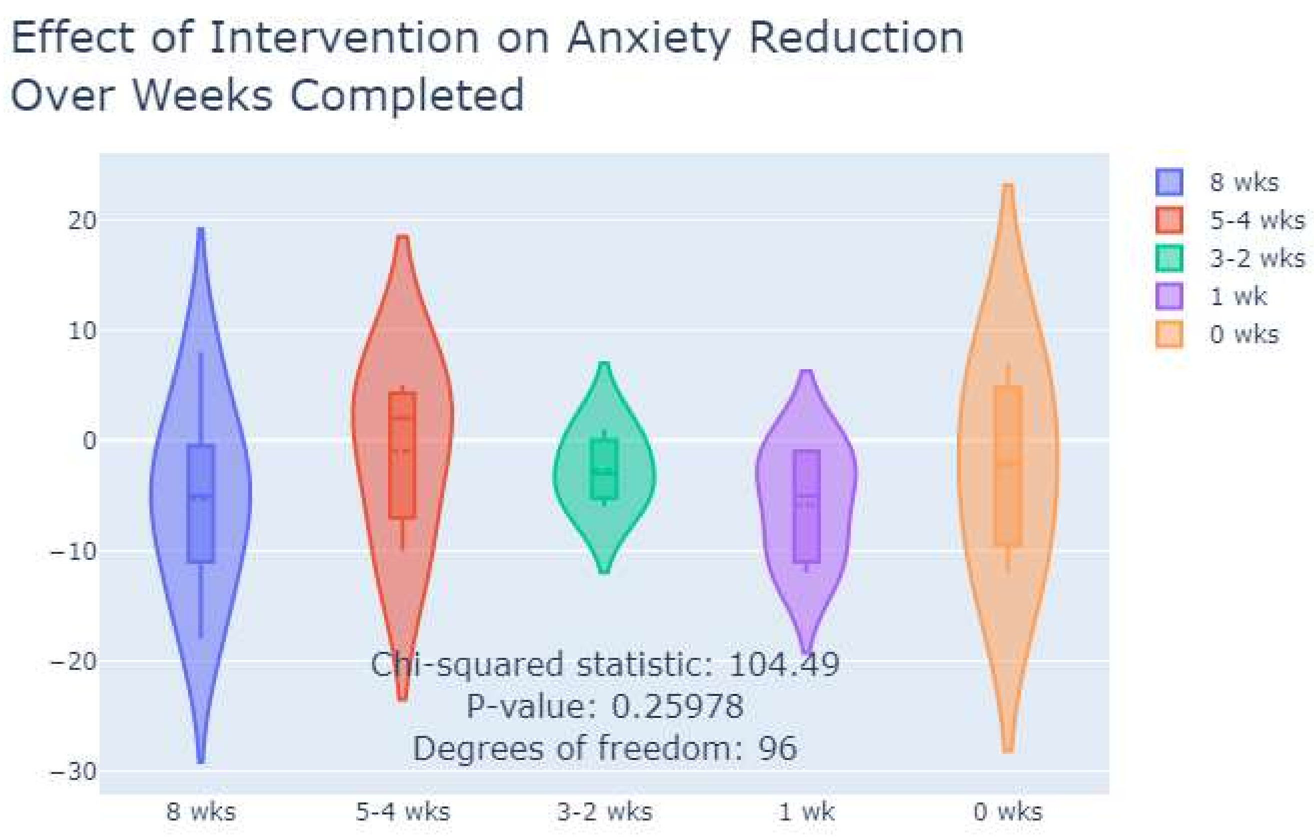


In [44]:
```
url = 'https://raw.githubusercontent.com/lauricocha/Digital-Art-Making-Research-2024/main/MFoA-data.csv'
MFOA = pd.read_csv(url)

correlation = MFOA['Weeks_Completed'].corr(MFOA['STAI-change'])
```

In [45]:
```
# Check the correlation
if correlation == 0:
    print("There is no linear correlation between the two columns.")
else:
    print(f"There is a correlation of {correlation:.2f} between the two columns.")
```

```
There is a correlation of -0.03 between the two columns.
```

In [46]:
```
from scipy.stats import pearsonr
correlation, p_value = pearsonr(MFOA['Weeks_Completed'], MFOA['STAI-change'])
```

In [47]:
```
correlation_coefficient, p_value = pearsonr(MFOA['Weeks_Completed'], MFOA['STAI-change'])

# Output the results
print(f"Pearson correlation coefficient (r): {correlation_coefficient}")
print(f"P-value: {p_value}")
```

```
Pearson correlation coefficient (r): -0.03344503546661931
P-value: 0.8825361056947121
```

In [48]:
```
contingency_table = pd.crosstab(MFOA['Weeks_Completed'], MFOA['STAI-change'])
```

In [49]:
```python
from scipy.stats import chi2_contingency

chi2, p, dof, expected = chi2_contingency(contingency_table)

# Output the results Figure 4A
print(f"Chi-squared statistic: {chi2}")
print(f"P-value: {p}")
print(f"Degrees of freedom: {dof}")
print("Expected frequencies:")
print(expected)
```

```
Chi-squared statistic: 104.49999999999999
P-value: 0.2597868856347276
Degrees of freedom: 96
Expected frequencies:
[[0.13636364 0.40909091 0.13636364 0.13636364 0.13636364 0.13636364
  0.13636364 0.13636364 0.27272727 0.27272727 0.27272727 0.13636364
  0.13636364 0.13636364 0.13636364 0.13636364 0.13636364]
 [0.27272727 0.81818182 0.27272727 0.27272727 0.27272727 0.27272727
  0.27272727 0.27272727 0.54545455 0.54545455 0.54545455 0.27272727
  0.27272727 0.27272727 0.27272727 0.27272727 0.27272727]
 [0.09090909 0.27272727 0.09090909 0.09090909 0.09090909 0.09090909
  0.09090909 0.09090909 0.18181818 0.18181818 0.18181818 0.09090909
  0.09090909 0.09090909 0.09090909 0.09090909 0.09090909]
 [0.04545455 0.13636364 0.04545455 0.04545455 0.04545455 0.04545455
  0.04545455 0.04545455 0.09090909 0.09090909 0.09090909 0.04545455
  0.04545455 0.04545455 0.04545455 0.04545455 0.04545455]
 [0.04545455 0.13636364 0.04545455 0.04545455 0.04545455 0.04545455
  0.04545455 0.04545455 0.09090909 0.09090909 0.09090909 0.04545455
  0.04545455 0.04545455 0.04545455 0.04545455 0.04545455]
 [0.09090909 0.27272727 0.09090909 0.09090909 0.09090909 0.09090909
  0.09090909 0.09090909 0.18181818 0.18181818 0.18181818 0.09090909
  0.09090909 0.09090909 0.09090909 0.09090909 0.09090909]
 [0.31818182 0.95454545 0.31818182 0.31818182 0.31818182 0.31818182
  0.31818182 0.31818182 0.63636364 0.63636364 0.63636364 0.31818182
  0.31818182 0.31818182 0.31818182 0.31818182 0.31818182]]
```

In [50]:
```python
cols = ['Initial_Anxiety_level','STAI-change','Weeks_done']
ComValue = df[cols]
```

In [51]:
```python
ComValue
```

Out[51]:

| | Initial_Anxiety_level | STAI-change | Weeks_done |
|---|---|---|---|
| **0** | low_anxiety | 8 | 8 wks |
| **1** | low_anxiety | -2 | 8 wks |
| **2** | moderate_anxiety | -1 | 1 wk |
| **3** | high_anxiety | -5 | 8 wks |
| **4** | high_anxiety | -12 | 8 wks |
| **5** | high_anxiety | -18 | 8 wks |
| **6** | moderate_anxiety | 0 | 8 wks |
| **7** | low_anxiety | -8 | 8 wks |
| **8** | low_anxiety | 5 | 5-4 wks |
| **9** | low_anxiety | 2 | 5-4 wks |
| **10** | high_anxiety | -10 | 5-4 wks |
| **11** | low_anxiety | -3 | 3-2 wks |
| **12** | low_anxiety | 1 | 3-2 wks |
| **13** | low_anxiety | -6 | 3-2 wks |
| **14** | low_anxiety | -1 | 1 wk |
| **15** | high_anxiety | -7 | 1 wk |
| **16** | high_anxiety | -11 | 1 wk |
| **17** | high_anxiety | -12 | 0 wks |
| **18** | moderate_anxiety | -12 | 1 wk |
| **19** | high_anxiety | -2 | 0 wks |
| **20** | moderate_anxiety | -3 | 1 wk |
| **21** | high_anxiety | 7 | 0 wks |

In [52]:
```python
df2 = ComValue.loc[(df['Weeks_done'] !='Zero')]
df2
```

Out[52]:

| | Initial_Anxiety_level | STAI-change | Weeks_done |
|---|---|---|---|
| **0** | low_anxiety | 8 | 8 wks |
| **1** | low_anxiety | -2 | 8 wks |
| **2** | moderate_anxiety | -1 | 1 wk |
| **3** | high_anxiety | -5 | 8 wks |
| **4** | high_anxiety | -12 | 8 wks |
| **5** | high_anxiety | -18 | 8 wks |
| **6** | moderate_anxiety | 0 | 8 wks |
| **7** | low_anxiety | -8 | 8 wks |
| **8** | low_anxiety | 5 | 5-4 wks |
| **9** | low_anxiety | 2 | 5-4 wks |
| **10** | high_anxiety | -10 | 5-4 wks |
| **11** | low_anxiety | -3 | 3-2 wks |
| **12** | low_anxiety | 1 | 3-2 wks |
| **13** | low_anxiety | -6 | 3-2 wks |
| **14** | low_anxiety | -1 | 1 wk |
| **15** | high_anxiety | -7 | 1 wk |
| **16** | high_anxiety | -11 | 1 wk |
| **17** | high_anxiety | -12 | 0 wks |
| **18** | moderate_anxiety | -12 | 1 wk |
| **19** | high_anxiety | -2 | 0 wks |
| **20** | moderate_anxiety | -3 | 1 wk |
| **21** | high_anxiety | 7 | 0 wks |

In [53]:

```
columns_to_drop = ['Age', 'STAI-T0', 'STAI-T1', 'Liking', 'Application', 'Gender', 'Ethnicity ID', 'Weeks_done','VAIAK', 'SSCS', 'Final_Anx
Anxiety = MFOA.drop(columns_to_drop, axis=1)

Anxiety
```

Out[53]:

| | STAI-change | Weeks_Completed |
|---|---|---|
| **0** | 8 | 8 |
| **1** | -2 | 8 |
| **2** | -1 | 1 |
| **3** | -5 | 8 |
| **4** | -12 | 8 |
| **5** | -18 | 8 |
| **6** | 0 | 8 |
| **7** | -8 | 8 |
| **8** | 5 | 5 |
| **9** | 2 | 5 |
| **10** | -10 | 4 |
| **11** | -3 | 3 |
| **12** | 1 | 2 |
| **13** | -6 | 2 |
| **14** | -1 | 1 |
| **15** | -7 | 1 |
| **16** | -11 | 1 |
| **17** | -12 | 0 |
| **18** | -12 | 1 |
| **19** | -2 | 0 |
| **20** | -3 | 1 |
| **21** | 7 | 0 |

In [54]:

```
Anxiety.corr()
```

Out[54]:

| | STAI-change | Weeks_Completed |
|---|---|---|
| **STAI-change** | 1.000000 | -0.033445 |
| **Weeks_Completed** | -0.033445 | 1.000000 |

In [55]:

```
corr = Anxiety.corr().round(2)

# plot the heatmap
plt.figure(figsize = (8,6))

#viz the heatmap
sns.heatmap(corr, annot= True)
```

Out[55]:

```
<Axes: >
```

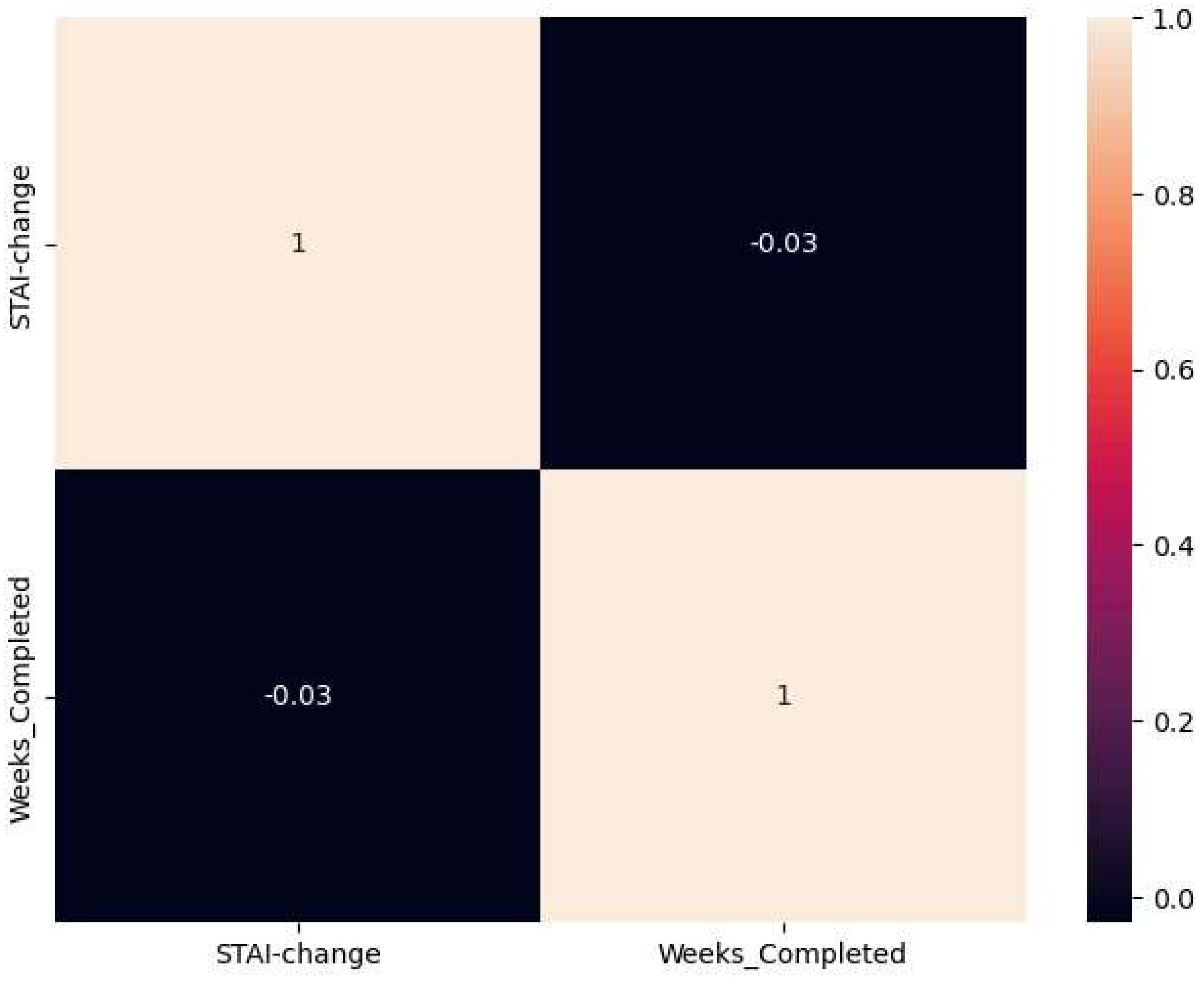


# Effect of intervention on lowering anxiety across various initial anxiety levels

In [57]:

```
import plotly.graph_objects as go
import pandas as pd
import plotly.io as pio
from IPython.display import Image

# Read the CSV file
url = 'https://raw.githubusercontent.com/lauricocha/Digital-Art-Making-Research-2024/main/MFoA-data.csv'
df = pd.read_csv(url)

fig = go.Figure()

Anxiety_levels = ['low_anxiety', 'moderate_anxiety', 'high_anxiety']

for level in Anxiety_levels:
    fig.add_trace(go.Violin(x=df['Initial_Anxiety_level'][df['Initial_Anxiety_level'] == level],
                            y=df['STAI-change'][df['Initial_Anxiety_level'] == level],
                            name=level,
                            box_visible=True,
                            meanline_visible=True))

fig.update_layout(
    title="Effect of Intervention on Lowering Anxiety Across Various Initial Anxiety Levels",
    title_font=dict(size=22)
)

fig.add_annotation(
    text="Chi-squared statistic: 14.51<br>P-value: 0.00582<br>Degrees of freedom: 4",
    x=0.2,  # Adjust the x and y values for proper positioning
    y=-22,
    showarrow=False,
    font=dict(size=16)  # Adjust the font size as needed
)
```

```
# Save the figure as a static image using kaleido
pio.write_image(fig, "plotly_figure_anxiety_levels.png")

# Display the static image
display(Image("plotly_figure_anxiety_levels.png"))
```

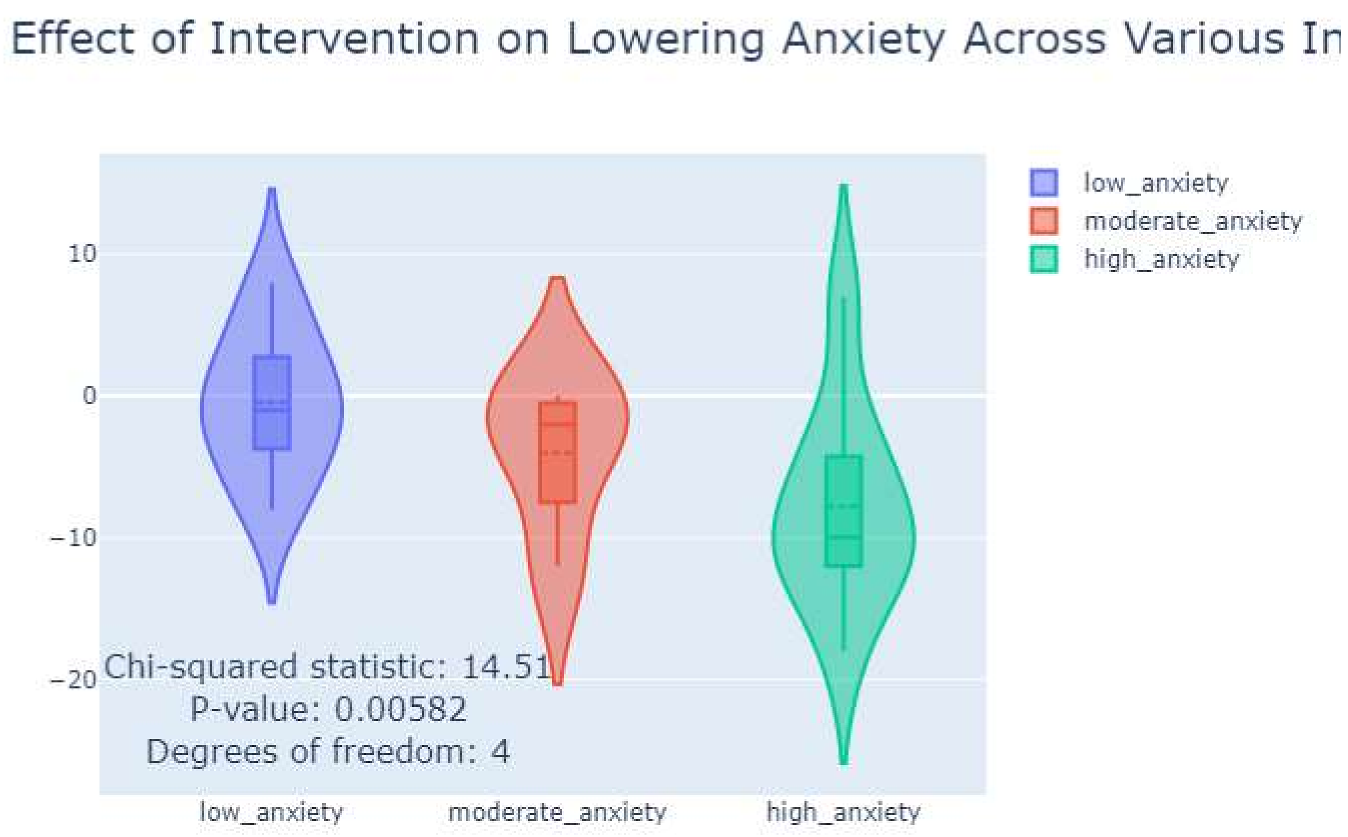


In [58]:
```
contingency_table = pd.crosstab(WeeklyV2['Pre_Intervention'], WeeklyV2['Post_Intervention'])
```

In [59]:
```
from scipy.stats import chi2_contingency

chi2, p, dof, expected = chi2_contingency(contingency_table)

# Output the results Figure 4B
print(f"Chi-squared statistic: {chi2}")
print(f"P-value: {p}")
print(f"Degrees of freedom: {dof}")
print("Expected frequencies:")
print(expected)
```

```
Chi-squared statistic: 14.51388888888889
P-value: 0.005823290218549798
Degrees of freedom: 4
Expected frequencies:
[[5.68421053 1.89473684 1.42105263]
 [2.52631579 0.84210526 0.63157895]
 [3.78947368 1.26315789 0.94736842]]
```

In [60]:
```
from scipy.stats import pearsonr
correlation, p_value = pearsonr(WeeklyV2['Pre_Intervention'], WeeklyV2['Post_Intervention'])
```

In [61]:
```
correlation_coefficient, p_value = pearsonr(WeeklyV2['Pre_Intervention'], WeeklyV2['Post_Intervention'])

# Output the results Figure 4B
print(f"Pearson correlation coefficient (r): {correlation_coefficient}")
print(f"P-value: {p_value}")
```

```
Pearson correlation coefficient (r): 0.7670113017599967
P-value: 0.00012722115037199815
```

# Extra data analysis

In [63]:
```
cols = ['VAIAK','STAI-change','Weeks_done']
ComValue = MFOA[cols]
```

In [64]:
```
ComValue
```

Out[64]:

| | VAIAK | STAI-change | Weeks_done |
|---|---|---|---|
| 0 | 53 | 8 | 8 wks |
| 1 | 37 | -2 | 8 wks |
| 2 | 19 | -1 | 1 wk |
| 3 | 21 | -5 | 8 wks |
| 4 | 25 | -12 | 8 wks |
| 5 | 45 | -18 | 8 wks |
| 6 | 39 | 0 | 8 wks |
| 7 | 33 | -8 | 8 wks |
| 8 | 44 | 5 | 5-4 wks |
| 9 | 43 | 2 | 5-4 wks |
| 10 | 20 | -10 | 5-4 wks |
| 11 | 42 | -3 | 3-2 wks |
| 12 | 41 | 1 | 3-2 wks |
| 13 | 30 | -6 | 3-2 wks |
| 14 | 25 | -1 | 1 wk |
| 15 | 55 | -7 | 1 wk |
| 16 | 49 | -11 | 1 wk |
| 17 | 44 | -12 | 0 wks |
| 18 | 42 | -12 | 1 wk |
| 19 | 53 | -2 | 0 wks |
| 20 | 52 | -3 | 1 wk |
| 21 | 55 | 7 | 0 wks |

In [65]:

```
df3 = ComValue.loc[(MFOA['Weeks_done'] !='Zero')]
df3
```

Out[65]:

| | VAIAK | STAI-change | Weeks_done |
|---|---|---|---|
| 0 | 53 | 8 | 8 wks |
| 1 | 37 | -2 | 8 wks |
| 2 | 19 | -1 | 1 wk |
| 3 | 21 | -5 | 8 wks |
| 4 | 25 | -12 | 8 wks |
| 5 | 45 | -18 | 8 wks |
| 6 | 39 | 0 | 8 wks |
| 7 | 33 | -8 | 8 wks |
| 8 | 44 | 5 | 5-4 wks |
| 9 | 43 | 2 | 5-4 wks |
| 10 | 20 | -10 | 5-4 wks |
| 11 | 42 | -3 | 3-2 wks |
| 12 | 41 | 1 | 3-2 wks |
| 13 | 30 | -6 | 3-2 wks |
| 14 | 25 | -1 | 1 wk |
| 15 | 55 | -7 | 1 wk |
| 16 | 49 | -11 | 1 wk |
| 17 | 44 | -12 | 0 wks |
| 18 | 42 | -12 | 1 wk |
| 19 | 53 | -2 | 0 wks |
| 20 | 52 | -3 | 1 wk |
| 21 | 55 | 7 | 0 wks |

In [66]:

```
df3.describe()
```

Out[66]:

| | VAIAK | STAI-change |
|---|---|---|
| **count** | 22.000000 | 22.000000 |
| **mean** | 39.409091 | -4.090909 |
| **std** | 11.733790 | 6.739430 |
| **min** | 19.000000 | -18.000000 |
| **25%** | 30.750000 | -9.500000 |
| **50%** | 42.000000 | -3.000000 |
| **75%** | 48.000000 | -0.250000 |
| **max** | 55.000000 | 8.000000 |

In [67]:
```
contingency_table = pd.crosstab(df3['VAIAK'], df3['STAI-change'])
```

In [68]:
```
from scipy.stats import chi2_contingency

chi2, p, dof, expected = chi2_contingency(contingency_table)

# Output the results
print(f"Chi-squared statistic: {chi2}")
print(f"P-value: {p}")
print(f"Degrees of freedom: {dof}")
print("Expected frequencies:")
print(expected)
```

```
Chi-squared statistic: 280.5
P-value: 0.14023478724909033
Degrees of freedom: 256
Expected frequencies:
[[0.04545455 0.13636364 0.04545455 0.04545455 0.04545455 0.04545455
  0.04545455 0.04545455 0.09090909 0.09090909 0.09090909 0.04545455
  0.04545455 0.04545455 0.04545455 0.04545455 0.04545455]
 [0.04545455 0.13636364 0.04545455 0.04545455 0.04545455 0.04545455
  0.04545455 0.04545455 0.09090909 0.09090909 0.09090909 0.04545455
  0.04545455 0.04545455 0.04545455 0.04545455 0.04545455]
 [0.04545455 0.13636364 0.04545455 0.04545455 0.04545455 0.04545455
  0.04545455 0.04545455 0.09090909 0.09090909 0.09090909 0.04545455
  0.04545455 0.04545455 0.04545455 0.04545455 0.04545455]
 [0.09090909 0.27272727 0.09090909 0.09090909 0.09090909 0.09090909
  0.09090909 0.09090909 0.18181818 0.18181818 0.18181818 0.09090909
  0.09090909 0.09090909 0.09090909 0.09090909 0.09090909]
 [0.04545455 0.13636364 0.04545455 0.04545455 0.04545455 0.04545455
  0.04545455 0.04545455 0.09090909 0.09090909 0.09090909 0.04545455
  0.04545455 0.04545455 0.04545455 0.04545455 0.04545455]
 [0.04545455 0.13636364 0.04545455 0.04545455 0.04545455 0.04545455
  0.04545455 0.04545455 0.09090909 0.09090909 0.09090909 0.04545455
  0.04545455 0.04545455 0.04545455 0.04545455 0.04545455]
 [0.04545455 0.13636364 0.04545455 0.04545455 0.04545455 0.04545455
  0.04545455 0.04545455 0.09090909 0.09090909 0.09090909 0.04545455
  0.04545455 0.04545455 0.04545455 0.04545455 0.04545455]
 [0.04545455 0.13636364 0.04545455 0.04545455 0.04545455 0.04545455
  0.04545455 0.04545455 0.09090909 0.09090909 0.09090909 0.04545455
  0.04545455 0.04545455 0.04545455 0.04545455 0.04545455]
 [0.04545455 0.13636364 0.04545455 0.04545455 0.04545455 0.04545455
  0.04545455 0.04545455 0.09090909 0.09090909 0.09090909 0.04545455
  0.04545455 0.04545455 0.04545455 0.04545455 0.04545455]
 [0.09090909 0.27272727 0.09090909 0.09090909 0.09090909 0.09090909
  0.09090909 0.09090909 0.18181818 0.18181818 0.18181818 0.09090909
  0.09090909 0.09090909 0.09090909 0.09090909 0.09090909]
 [0.04545455 0.13636364 0.04545455 0.04545455 0.04545455 0.04545455
  0.04545455 0.04545455 0.09090909 0.09090909 0.09090909 0.04545455
  0.04545455 0.04545455 0.04545455 0.04545455 0.04545455]
 [0.09090909 0.27272727 0.09090909 0.09090909 0.09090909 0.09090909
  0.09090909 0.09090909 0.18181818 0.18181818 0.18181818 0.09090909
  0.09090909 0.09090909 0.09090909 0.09090909 0.09090909]
 [0.04545455 0.13636364 0.04545455 0.04545455 0.04545455 0.04545455
  0.04545455 0.04545455 0.09090909 0.09090909 0.09090909 0.04545455
  0.04545455 0.04545455 0.04545455 0.04545455 0.04545455]
 [0.04545455 0.13636364 0.04545455 0.04545455 0.04545455 0.04545455
  0.04545455 0.04545455 0.09090909 0.09090909 0.09090909 0.04545455
  0.04545455 0.04545455 0.04545455 0.04545455 0.04545455]
 [0.04545455 0.13636364 0.04545455 0.04545455 0.04545455 0.04545455
  0.04545455 0.04545455 0.09090909 0.09090909 0.09090909 0.04545455
  0.04545455 0.04545455 0.04545455 0.04545455 0.04545455]
 [0.09090909 0.27272727 0.09090909 0.09090909 0.09090909 0.09090909
  0.09090909 0.09090909 0.18181818 0.18181818 0.18181818 0.09090909
  0.09090909 0.09090909 0.09090909 0.09090909 0.09090909]
 [0.09090909 0.27272727 0.09090909 0.09090909 0.09090909 0.09090909
  0.09090909 0.09090909 0.18181818 0.18181818 0.18181818 0.09090909
  0.09090909 0.09090909 0.09090909 0.09090909 0.09090909]]
```

In [69]:
```
correlation = df3['VAIAK'].corr(df3['STAI-change'])
```

In [70]:
```
# Check the correlation
if correlation == 0:
```

```
    print("There is no linear correlation between the two columns.")
else:
    print(f"There is a correlation of {correlation:.2f} between the two columns.")
```

There is a correlation of 0.23 between the two columns.

In [71]:
```
from scipy.stats import pearsonr
correlation, p_value = pearsonr(df3['VAIAK'], df3['STAI-change'])
```

In [72]:
```
correlation_coefficient, p_value = pearsonr(df3['VAIAK'], df3['STAI-change'])

# Output the results
print(f"Pearson correlation coefficient (r): {correlation_coefficient}")
print(f"P-value: {p_value}")
```

Pearson correlation coefficient (r): 0.22991953012082614
P-value: 0.3033189838830542

In [73]:
```
if p_value < 0.05:  # You can adjust the significance level (alpha) as needed
    print(f"There is a statistically significant correlation of {correlation:.2f} between the two columns.")
else:
    print(f"There is no statistically significant correlation between the two columns.")
```

There is no statistically significant correlation between the two columns.

In [74]:
```
url2 = 'https://raw.githubusercontent.com/lauricocha/Digital-Art-Making-Research-2024/main/MFoA-data.csv'
df = pd.read_csv(url2)
```

In [75]:
```
cols = ['SSCS','STAI-change','Weeks_done']
ComValue = MFOA[cols]
```

In [76]:
```
ComValue
```

Out[76]:

| | SSCS | STAI-change | Weeks_done |
|---|---|---|---|
| **0** | 39 | 8 | 8 wks |
| **1** | 36 | -2 | 8 wks |
| **2** | 28 | -1 | 1 wk |
| **3** | 23 | -5 | 8 wks |
| **4** | 31 | -12 | 8 wks |
| **5** | 38 | -18 | 8 wks |
| **6** | 31 | 0 | 8 wks |
| **7** | 42 | -8 | 8 wks |
| **8** | 42 | 5 | 5-4 wks |
| **9** | 26 | 2 | 5-4 wks |
| **10** | 44 | -10 | 5-4 wks |
| **11** | 37 | -3 | 3-2 wks |
| **12** | 35 | 1 | 3-2 wks |
| **13** | 28 | -6 | 3-2 wks |
| **14** | 42 | -1 | 1 wk |
| **15** | 36 | -7 | 1 wk |
| **16** | 36 | -11 | 1 wk |
| **17** | 31 | -12 | 0 wks |
| **18** | 38 | -12 | 1 wk |
| **19** | 39 | -2 | 0 wks |
| **20** | 37 | -3 | 1 wk |
| **21** | 33 | 7 | 0 wks |

In [77]:
```
df4 = ComValue.loc[(MFOA['Weeks_done'] !='Zero')]
df4
```

Out[77]:

| | SSCS | STAI-change | Weeks_done |
|---|---|---|---|
| **0** | 39 | 8 | 8 wks |
| **1** | 36 | -2 | 8 wks |
| **2** | 28 | -1 | 1 wk |
| **3** | 23 | -5 | 8 wks |
| **4** | 31 | -12 | 8 wks |
| **5** | 38 | -18 | 8 wks |
| **6** | 31 | 0 | 8 wks |
| **7** | 42 | -8 | 8 wks |
| **8** | 42 | 5 | 5-4 wks |
| **9** | 26 | 2 | 5-4 wks |
| **10** | 44 | -10 | 5-4 wks |
| **11** | 37 | -3 | 3-2 wks |
| **12** | 35 | 1 | 3-2 wks |
| **13** | 28 | -6 | 3-2 wks |
| **14** | 42 | -1 | 1 wk |
| **15** | 36 | -7 | 1 wk |
| **16** | 36 | -11 | 1 wk |
| **17** | 31 | -12 | 0 wks |
| **18** | 38 | -12 | 1 wk |
| **19** | 39 | -2 | 0 wks |
| **20** | 37 | -3 | 1 wk |
| **21** | 33 | 7 | 0 wks |

In [78]:

```
contingency_table = pd.crosstab(df4['SSCS'], df4['STAI-change'])
```

In [79]:

```
from scipy.stats import chi2_contingency

chi2, p, dof, expected = chi2_contingency(contingency_table)

# Output the results
print(f"Chi-squared statistic: {chi2}")
print(f"P-value: {p}")
print(f"Degrees of freedom: {dof}")
print("Expected frequencies:")
print(expected)
```

```
Chi-squared statistic: 211.44444444444446
P-value: 0.035113519566501175
Degrees of freedom: 176
Expected frequencies:
[[0.04545455 0.13636364 0.04545455 0.04545455 0.04545455 0.04545455
  0.04545455 0.04545455 0.09090909 0.09090909 0.09090909 0.04545455
  0.04545455 0.04545455 0.04545455 0.04545455 0.04545455]
 [0.04545455 0.13636364 0.04545455 0.04545455 0.04545455 0.04545455
  0.04545455 0.04545455 0.09090909 0.09090909 0.09090909 0.04545455
  0.04545455 0.04545455 0.04545455 0.04545455 0.04545455]
 [0.09090909 0.27272727 0.09090909 0.09090909 0.09090909 0.09090909
  0.09090909 0.09090909 0.18181818 0.18181818 0.18181818 0.09090909
  0.09090909 0.09090909 0.09090909 0.09090909 0.09090909]
 [0.13636364 0.40909091 0.13636364 0.13636364 0.13636364 0.13636364
  0.13636364 0.13636364 0.27272727 0.27272727 0.27272727 0.13636364
  0.13636364 0.13636364 0.13636364 0.13636364 0.13636364]
 [0.04545455 0.13636364 0.04545455 0.04545455 0.04545455 0.04545455
  0.04545455 0.04545455 0.09090909 0.09090909 0.09090909 0.04545455
  0.04545455 0.04545455 0.04545455 0.04545455 0.04545455]
 [0.04545455 0.13636364 0.04545455 0.04545455 0.04545455 0.04545455
  0.04545455 0.04545455 0.09090909 0.09090909 0.09090909 0.04545455
  0.04545455 0.04545455 0.04545455 0.04545455 0.04545455]
 [0.13636364 0.40909091 0.13636364 0.13636364 0.13636364 0.13636364
  0.13636364 0.13636364 0.27272727 0.27272727 0.27272727 0.13636364
  0.13636364 0.13636364 0.13636364 0.13636364 0.13636364]
 [0.09090909 0.27272727 0.09090909 0.09090909 0.09090909 0.09090909
  0.09090909 0.09090909 0.18181818 0.18181818 0.18181818 0.09090909
  0.09090909 0.09090909 0.09090909 0.09090909 0.09090909]
 [0.09090909 0.27272727 0.09090909 0.09090909 0.09090909 0.09090909
  0.09090909 0.09090909 0.18181818 0.18181818 0.18181818 0.09090909
  0.09090909 0.09090909 0.09090909 0.09090909 0.09090909]
 [0.09090909 0.27272727 0.09090909 0.09090909 0.09090909 0.09090909
  0.09090909 0.09090909 0.18181818 0.18181818 0.18181818 0.09090909
  0.09090909 0.09090909 0.09090909 0.09090909 0.09090909]
 [0.13636364 0.40909091 0.13636364 0.13636364 0.13636364 0.13636364
  0.13636364 0.13636364 0.27272727 0.27272727 0.27272727 0.13636364
  0.13636364 0.13636364 0.13636364 0.13636364 0.13636364]
 [0.04545455 0.13636364 0.04545455 0.04545455 0.04545455 0.04545455
  0.04545455 0.04545455 0.09090909 0.09090909 0.09090909 0.04545455
  0.04545455 0.04545455 0.04545455 0.04545455 0.04545455]]
```

In [80]:
```python
correlation = df4['SSCS'].corr(df4['STAI-change'])
```

In [81]:
```python
# Check the correlation
if correlation == 0:
    print("There is no linear correlation between the two columns.")
else:
    print(f"There is a correlation of {correlation:.2f} between the two columns.")
```

```
There is a correlation of -0.04 between the two columns.
```

In [82]:
```python
from scipy.stats import pearsonr
correlation, p_value = pearsonr(df4['SSCS'], df4['STAI-change'])
```

In [83]:
```python
correlation_coefficient, p_value = pearsonr(df4['SSCS'], df4['STAI-change'])

# Output the results
print(f"Pearson correlation coefficient (r): {correlation_coefficient}")
print(f"P-value: {p_value}")
```

```
Pearson correlation coefficient (r): -0.04375711886852955
P-value: 0.8466841550578175
```

In [84]:
```python
if p_value < 0.05:  # You can adjust the significance level (alpha) as needed
    print(f"There is a statistically significant correlation of {correlation:.2f} between the two columns.")
else:
    print(f"There is no statistically significant correlation between the two columns.")
```

```
There is no statistically significant correlation between the two columns.
```

In [85]:
```python
df4.describe()
```

Out[85]:

| | SSCS | STAI-change |
|---|---|---|
| **count** | 22.000000 | 22.000000 |
| **mean** | 35.090909 | -4.090909 |
| **std** | 5.622312 | 6.739430 |
| **min** | 23.000000 | -18.000000 |
| **25%** | 31.000000 | -9.500000 |
| **50%** | 36.000000 | -3.000000 |
| **75%** | 38.750000 | -0.250000 |
| **max** | 44.000000 | 8.000000 |

In [86]:
```python
columns_to_drop = ['ID']  # Replace with the actual column names
STAI = Weekly.drop(columns_to_drop, axis=1)
```

```
STAI
```

Out[86]:

| | Week_number | STAI | FLOW | Arousal | Positivity |
|---|---|---|---|---|---|
| **0** | 0.0 | 29 | 0 | 0 | 0 |
| **1** | 1.0 | 28 | 44 | 5 | 9 |
| **2** | 2.0 | 27 | 42 | 7 | 9 |
| **3** | 3.0 | 33 | 40 | 3 | 9 |
| **4** | 4.0 | 26 | 43 | 3 | 9 |
| **...** | ... | ... | ... | ... | ... |
| **105** | 1.0 | 41 | 35 | 9 | 4 |
| **106** | 0.0 | 52 | 0 | 0 | 0 |
| **107** | 1.0 | 41 | 40 | 4 | 5 |
| **108** | 0.0 | 41 | 0 | 0 | 0 |
| **109** | 1.0 | 40 | 32 | 5 | 3 |

110 rows × 5 columns

In [87]:

```
STAI.corr()
```

Out[87]:

| | Week_number | STAI | FLOW | Arousal | Positivity |
|---|---|---|---|---|---|
| **Week_number** | 1.000000 | -0.227399 | 0.115557 | 0.022679 | 0.098003 |
| **STAI** | -0.227399 | 1.000000 | -0.319320 | -0.030192 | -0.470297 |
| **FLOW** | 0.115557 | -0.319320 | 1.000000 | 0.677257 | 0.912332 |
| **Arousal** | 0.022679 | -0.030192 | 0.677257 | 1.000000 | 0.591384 |
| **Positivity** | 0.098003 | -0.470297 | 0.912332 | 0.591384 | 1.000000 |

In [88]:

```
corr = STAI.corr().round(2)

# plot the heatmap
plt.figure(figsize = (15,10))

#viz the heatmap
sns.heatmap(corr, annot= True)
```

Out[88]: <Axes: >

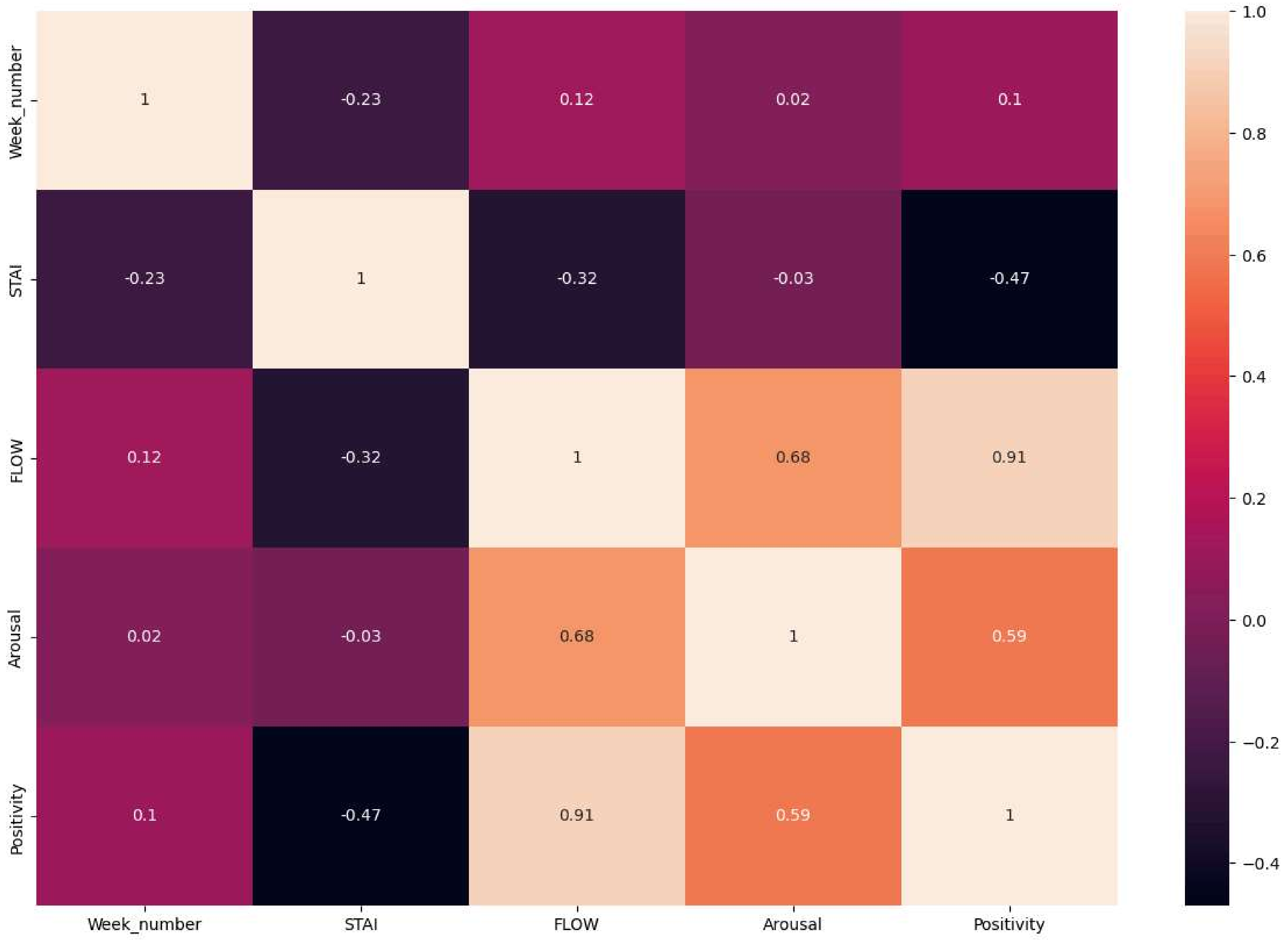


In [89]:

```
import numpy as np
import pandas as pd
import seaborn as sns
import scipy.stats as stats

# Calculate the correlation coefficients
correlation_matrix = STAI.corr()

# Calculate the p-values
p_values = STAI.apply(lambda x: STAI.apply(lambda y: stats.pearsonr(x, y)[1]))

# Create a heatmap of the p-values
pvalue_heatmap = sns.heatmap(p_values, annot=True, cmap='rocket', fmt='.2f')
```

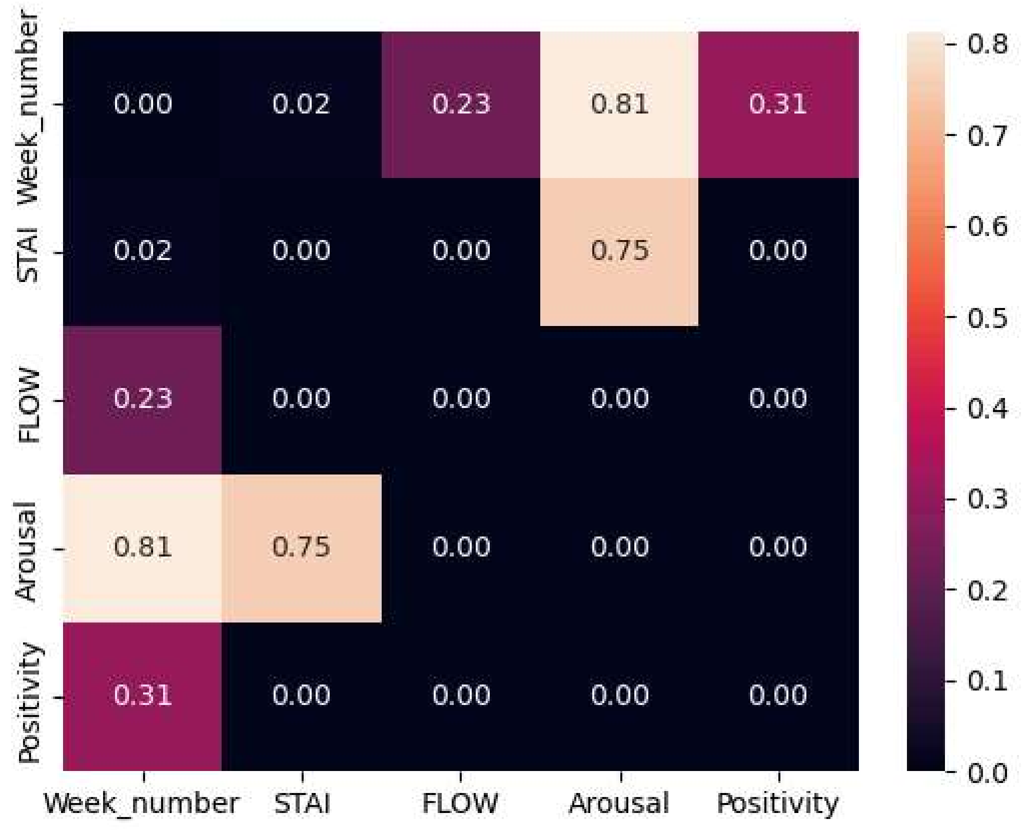


In [90]:

```
pvalue_heatmap.figure.savefig('heatmap.png', format='png', dpi=300, bbox_inches='tight')
```

In [91]:

```
# Define a custom color palette (you can replace these with your desired colors)
custom_palette = ["#7c1c5b"]
```

```
# Set the custom color palette
sns.set_palette(custom_palette)
ax = corr['Week_number'].sort_values()[:-1].plot(kind = 'bar', figsize = (10,6))
ax.set_title('Spearman Correlation of Weekly Survey Results', loc='left', y=1.05)

ax.bar_label(ax.containers[0]);
```

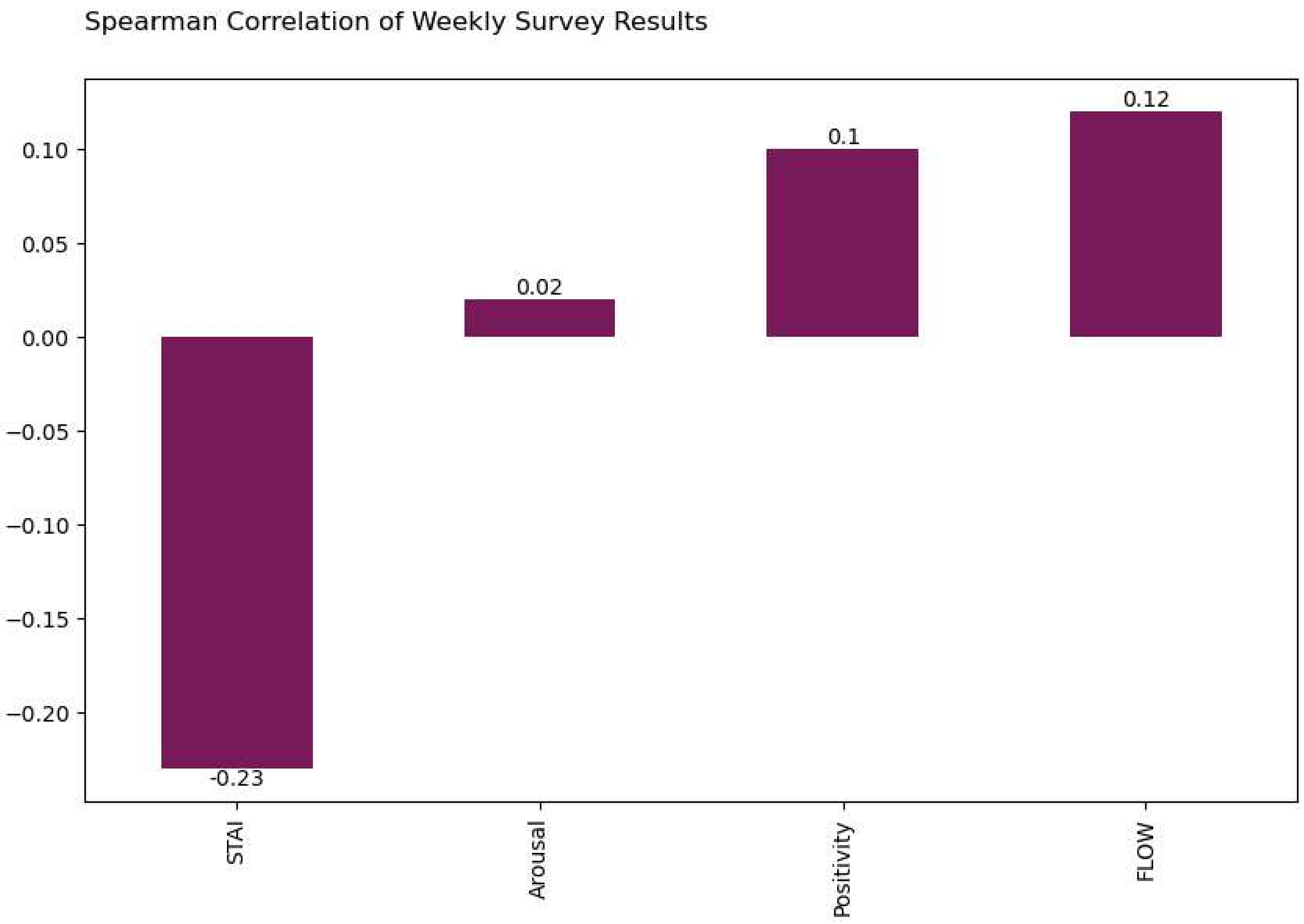


In [92]:

```
import numpy as np

# Example demographic and measurement data
demographic_data = {
    'Age': [52,23,20,72,24,28,57,32,26,28,50,51,61,25,19,31,56,31,53,24,25,23],
    'Gender': ['Female','Non-binary','Female','Female','Female','Male','Male','Female','Female','Female','Male','Female','Female','Male','P
    'Ethnicity': ['HispLat','White','Asian','White','White','White','White','Asian','HispLat','Mixed','Mixed','White','Other','HispLat','Hi
}


measurement_data = [8,-2,1,-5,-12,-18,5,0,-6,-3,-10,-8,-12,-12,-2,-3,7]

# Calculate Mean (Mage), Standard Deviation (SD), and Range
mage = np.mean(np.abs(np.array(measurement_data)))
sd = np.std(measurement_data)
data_range = np.ptp(measurement_data)

print("Mean Absolute Gross Error (Mage):", mage)
print("Standard Deviation (SD):", sd)
print("Range:", data_range)
```

```
Mean Absolute Gross Error (Mage): 6.705882352941177
Standard Deviation (SD): 7.033779101088303
Range: 26
```

In [42]:

```
contingency_table = pd.crosstab(WeeklyV2['Initial_STAI'], WeeklyV2['Final_STAI'])
```

In [43]:

```
from scipy.stats import chi2_contingency

chi2, p, dof, expected = chi2_contingency(contingency_table)


print(f"Chi-squared statistic: {chi2}")
print(f"P-value: {p}")
print(f"Degrees of freedom: {dof}")
print("Expected frequencies:")
print(expected)
```

```
Chi-squared statistic: 207.4166666666667
P-value: 0.2580196422944555
Degrees of freedom: 195
Expected frequencies:
[[0.05263158 0.05263158 0.10526316 0.10526316 0.05263158 0.05263158
  0.10526316 0.05263158 0.05263158 0.10526316 0.10526316 0.05263158
  0.05263158 0.05263158]
 [0.05263158 0.05263158 0.10526316 0.10526316 0.05263158 0.05263158
  0.10526316 0.05263158 0.05263158 0.10526316 0.10526316 0.05263158
  0.05263158 0.05263158]
 [0.05263158 0.05263158 0.10526316 0.10526316 0.05263158 0.05263158
  0.10526316 0.05263158 0.05263158 0.10526316 0.10526316 0.05263158
  0.05263158 0.05263158]
 [0.05263158 0.05263158 0.10526316 0.10526316 0.05263158 0.05263158
  0.10526316 0.05263158 0.05263158 0.10526316 0.10526316 0.05263158
  0.05263158 0.05263158]
 [0.10526316 0.10526316 0.21052632 0.21052632 0.10526316 0.10526316
  0.21052632 0.10526316 0.10526316 0.21052632 0.21052632 0.10526316
  0.10526316 0.10526316]
 [0.05263158 0.05263158 0.10526316 0.10526316 0.05263158 0.05263158
  0.10526316 0.05263158 0.05263158 0.10526316 0.10526316 0.05263158
  0.05263158 0.05263158]
 [0.05263158 0.05263158 0.10526316 0.10526316 0.05263158 0.05263158
  0.10526316 0.05263158 0.05263158 0.10526316 0.10526316 0.05263158
  0.05263158 0.05263158]
 [0.05263158 0.05263158 0.10526316 0.10526316 0.05263158 0.05263158
  0.10526316 0.05263158 0.05263158 0.10526316 0.10526316 0.05263158
  0.05263158 0.05263158]
 [0.05263158 0.05263158 0.10526316 0.10526316 0.05263158 0.05263158
  0.10526316 0.05263158 0.05263158 0.10526316 0.10526316 0.05263158
  0.05263158 0.05263158]
 [0.05263158 0.05263158 0.10526316 0.10526316 0.05263158 0.05263158
  0.10526316 0.05263158 0.05263158 0.10526316 0.10526316 0.05263158
  0.05263158 0.05263158]
 [0.05263158 0.05263158 0.10526316 0.10526316 0.05263158 0.05263158
  0.10526316 0.05263158 0.05263158 0.10526316 0.10526316 0.05263158
  0.05263158 0.05263158]
 [0.05263158 0.05263158 0.10526316 0.10526316 0.05263158 0.05263158
  0.10526316 0.05263158 0.05263158 0.10526316 0.10526316 0.05263158
  0.05263158 0.05263158]
 [0.05263158 0.05263158 0.10526316 0.10526316 0.05263158 0.05263158
  0.10526316 0.05263158 0.05263158 0.10526316 0.10526316 0.05263158
  0.05263158 0.05263158]
 [0.15789474 0.15789474 0.31578947 0.31578947 0.15789474 0.15789474
  0.31578947 0.15789474 0.15789474 0.31578947 0.31578947 0.15789474
  0.15789474 0.15789474]
 [0.05263158 0.05263158 0.10526316 0.10526316 0.05263158 0.05263158
  0.10526316 0.05263158 0.05263158 0.10526316 0.10526316 0.05263158
  0.05263158 0.05263158]
 [0.05263158 0.05263158 0.10526316 0.10526316 0.05263158 0.05263158
  0.10526316 0.05263158 0.05263158 0.10526316 0.10526316 0.05263158
  0.05263158 0.05263158]]
```

In [93]:
```python
url2 = 'https://raw.githubusercontent.com/lauricocha/Digital-Art-Making-Research-2024/main/MFoA-data-weekly.csv'
df4 = pd.read_csv(url2)


# Check for NaN values
print("NaN values:\n", df4.isnull().sum())
```

```
NaN values:
 Week_number    0
ID             0
STAI           0
FLOW           0
Arousal        0
Positivity     0
dtype: int64
```

In [94]:
```python
# Check data types of each column
print("Data Types:\n", df4.dtypes)
```

```
Data Types:
 Week_number    float64
ID              object
STAI             int64
FLOW             int64
Arousal          int64
Positivity       int64
dtype: object
```

In [95]:
```python
# Convert data types of numeric columns to float64
numeric_columns = ['STAI', 'FLOW', 'Arousal', 'Positivity']
df4[numeric_columns] = df4[numeric_columns].astype(float)

# Check data types after conversion
print("Updated Data Types:\n", df4.dtypes)

# Handle non-numeric values (e.g., convert 'inf' values to NaN)
df4.replace([np.inf, -np.inf], np.nan, inplace=True)

# Apply np.isinf() only to numeric columns
```

```
inf_values = df4[numeric_columns].apply(lambda x: np.isinf(x)).sum()

# Print the number of infinity values in each column
print("Inf values after dropping:\n", inf_values)
```

```
Updated Data Types:
 Week_number    float64
ID              object
STAI           float64
FLOW           float64
Arousal        float64
Positivity     float64
dtype: object
Inf values after dropping:
 STAI          0
FLOW          0
Arousal       0
Positivity    0
dtype: int64
```

In [96]:

```
import statsmodels.api as sm
from statsmodels.formula.api import ols

# Assuming 'df' is your DataFrame containing the data
# 'dependent_variable' is the name of your dependent variable column
# 'independent_variables' is a list of names of your independent variables
# 'data' is the DataFrame containing your data

# Construct the formula string
formula = 'STAI ~ ' + ' + '.join(['ID', 'Week_number'])

# Fit the model
model = ols(formula, data=df4).fit()

# Perform ANOVA
anova_table = sm.stats.anova_lm(model, typ=3)

# Print the ANOVA table with Type III sum of squares
print(anova_table)
```

```
                 sum_sq    df           F        PR(>F)
Intercept    8398.336756   1.0  131.153718  2.135719e-19
ID           5574.708570  16.0    5.441135  5.811043e-08
Week_number   477.925173   1.0    7.463581  7.548276e-03
Residual     5891.155779  92.0         NaN           NaN
```

In [97]:

```
import statsmodels.api as sm
from statsmodels.formula.api import ols

# Assuming 'df' is your DataFrame containing the data
# 'dependent_variable' is the name of your dependent variable column
# 'independent_variables' is a list of names of your independent variables
# 'data' is the DataFrame containing your data

# Construct the formula string
formula = 'STAI ~ ' + ' + '.join(['ID', 'Week_number'])

# Fit the model
model = ols(formula, data=df4).fit()

# Perform ANOVA
anova_table = sm.stats.anova_lm(model, typ=1)

# Print the ANOVA table with Type I sum of squares
print(anova_table)
```

```
               df       sum_sq     mean_sq         F        PR(>F)
ID           16.0  5722.019048  357.626190  5.584916  3.529800e-08
Week_number   1.0   477.925173  477.925173  7.463581  7.548276e-03
Residual     92.0  5891.155779   64.034302       NaN           NaN
```

# Extra data analysis

In [99]:

```
import plotly.express as px
color_scale_min = -18
color_scale_max = 8
df = px.data.wind()
fig = px.bar_polar(MFOA, r="VAIAK", theta="ID", color="STAI-change", range_color=[color_scale_min, color_scale_max],
                   template="plotly_dark",
                   color_continuous_scale= px.colors.sequential.Plasma_r,
                   title="Participant's Art Interest and Art Knowledge Effect on Art Intervention"
)
# Customize the title font size
fig.update_layout(title=dict(font=dict(size=24)))  # Adjust the size as needed

fig.show()
```

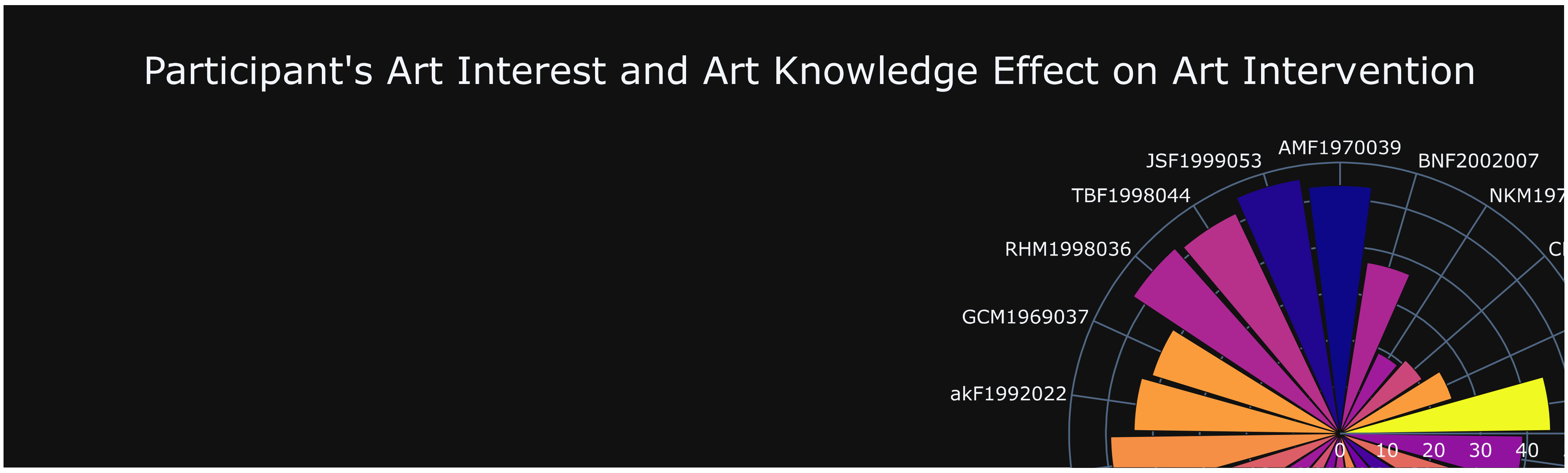


In [100…

```
import plotly.express as px
color_scale_min = -18
color_scale_max = 8
df = px.data.wind()
fig = px.bar_polar(MFOA, r="SSCS", theta="ID", color="STAI-change", range_color=[color_scale_min, color_scale_max],
                   template="plotly_dark",
                   color_continuous_scale=px.colors.sequential.Viridis,
                   title="Participant's Self-perceived levels of creativity Effect on Art Intervention"
)
# Customize the title font size
fig.update_layout(title=dict(font=dict(size=24)))  # Adjust the size as needed

fig.show()
```

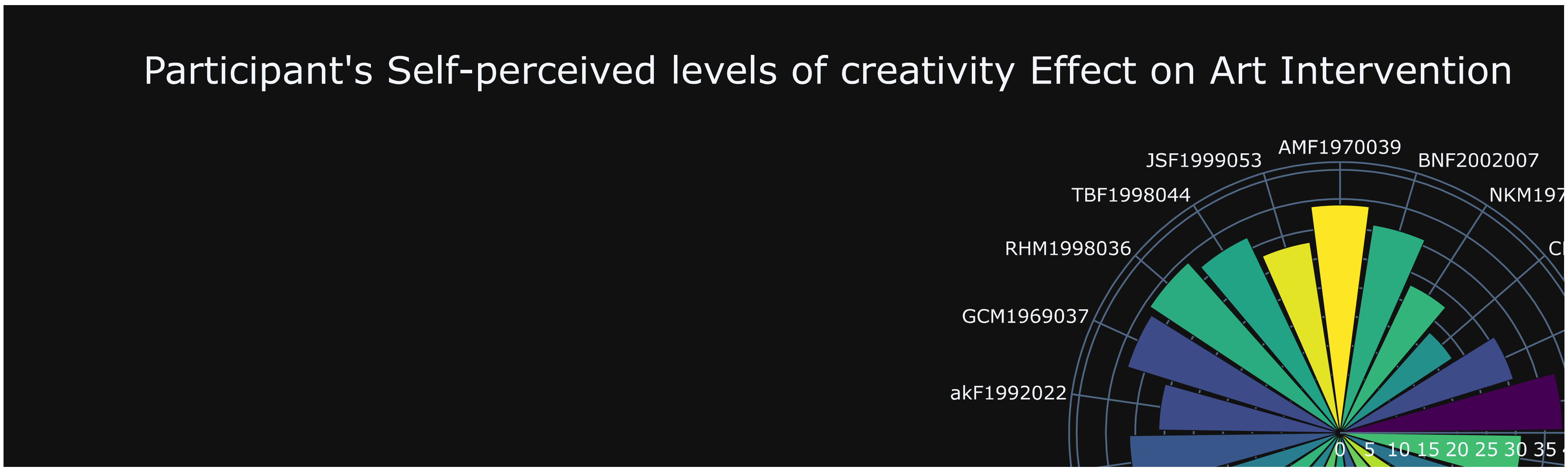


In [101…

```
Weekly.hist(figsize = (10, 8), grid = False, color= '#7c1c5b');
```

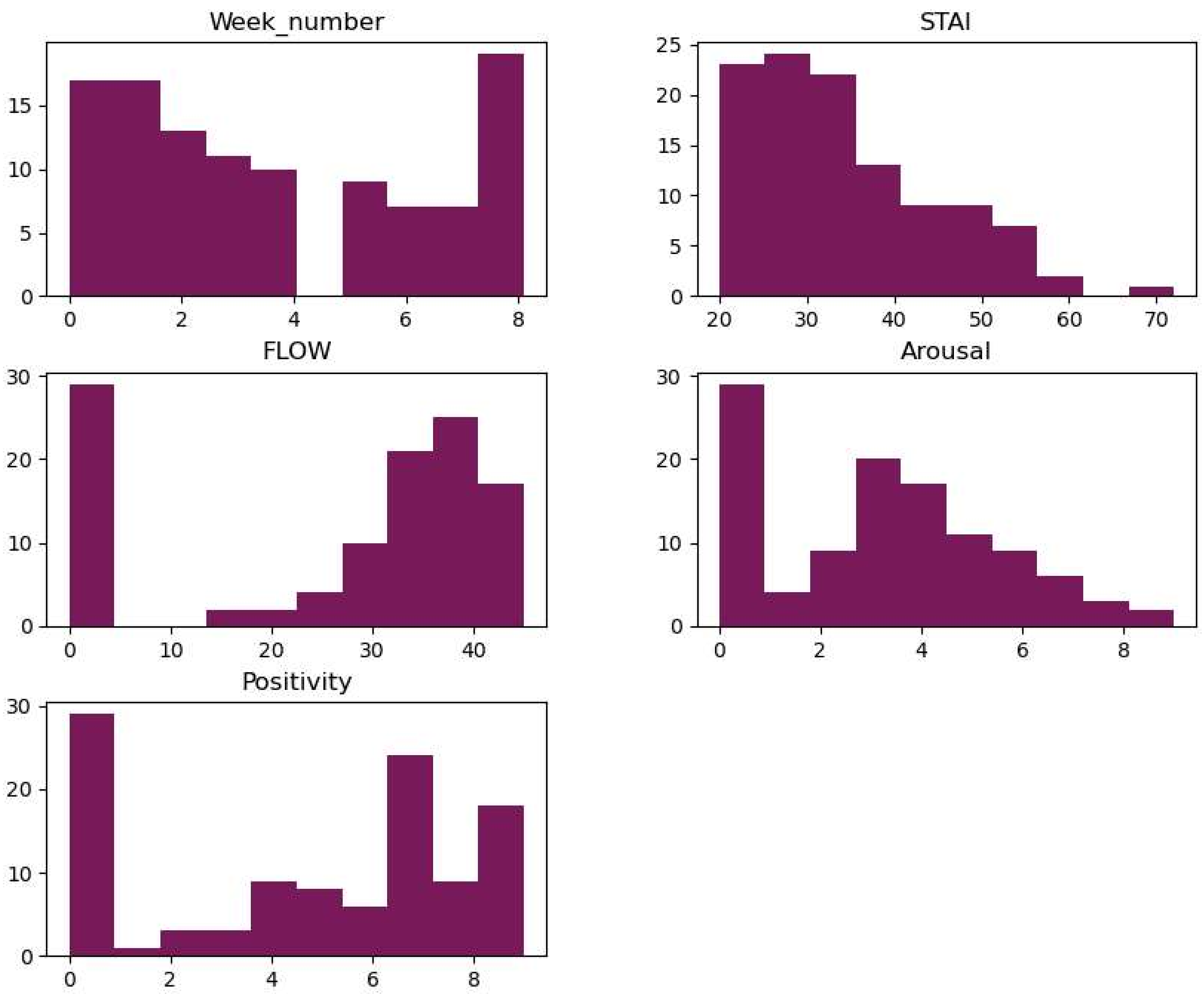


In [102…

```
pip install pandas plotly statsmodels
```

```
Requirement already satisfied: pandas in c:\users\vidalse\anaconda3\lib\site-packages (2.2.2)
Requirement already satisfied: plotly in c:\users\vidalse\anaconda3\lib\site-packages (5.22.0)
Requirement already satisfied: statsmodels in c:\users\vidalse\anaconda3\lib\site-packages (0.14.2)
Requirement already satisfied: numpy>=1.26.0 in c:\users\vidalse\anaconda3\lib\site-packages (from pandas) (1.26.4)
Requirement already satisfied: python-dateutil>=2.8.2 in c:\users\vidalse\anaconda3\lib\site-packages (from pandas) (2.9.0.post0)
Requirement already satisfied: pytz>=2020.1 in c:\users\vidalse\anaconda3\lib\site-packages (from pandas) (2024.1)
Requirement already satisfied: tzdata>=2022.7 in c:\users\vidalse\anaconda3\lib\site-packages (from pandas) (2023.3)
Requirement already satisfied: tenacity>=6.2.0 in c:\users\vidalse\anaconda3\lib\site-packages (from plotly) (8.2.2)
Requirement already satisfied: packaging in c:\users\vidalse\anaconda3\lib\site-packages (from plotly) (23.2)
Requirement already satisfied: scipy!=1.9.2,>=1.8 in c:\users\vidalse\anaconda3\lib\site-packages (from statsmodels) (1.13.1)
Requirement already satisfied: patsy>=0.5.6 in c:\users\vidalse\anaconda3\lib\site-packages (from statsmodels) (0.5.6)
Requirement already satisfied: six in c:\users\vidalse\anaconda3\lib\site-packages (from patsy>=0.5.6->statsmodels) (1.16.0)
Note: you may need to restart the kernel to use updated packages.
```

In [103…

```
url = 'https://raw.githubusercontent.com/lauricocha/Digital-Art-Making-Research-2024/main/MFoA-data.csv'
MFOA = pd.read_csv(url)
```

In [104…

```
MFOA.head(5)
```

Out[104…

| | ID | Age | Gender | Ethnicity ID | VAIAK | SSCS | Initial_Anxiety_level | STAI-T0 | STAI-T1 | Final_Anxiety_Level | STAI-change | Last_Recorded_STAI | Weeks_don |
|---|---|---|---|---|---|---|---|---|---|---|---|---|---|
| **0** | AMF1970039 | 52 | Female | HispLat | 53 | 39 | low_anxiety | 29 | 37.0 | low_anxiety_(20-37) | 8 | 37 | 8 wk |
| **1** | BNF2002007 | 20 | Female | Asian | 37 | 36 | low_anxiety | 25 | 23.0 | low_anxiety_(20-37) | -2 | 23 | 8 wk |
| **2** | NKM1972043 | 50 | Male | Mixed | 19 | 28 | moderate_anxiety | 41 | NaN | moderate_anxiety_(38-44) | -1 | 40 | 1 w |
| **3** | CDF1998025 | 24 | Female | White | 21 | 23 | high_anxiety | 52 | 47.0 | high_anxiety_(45-80) | -5 | 47 | 8 wk |
| **4** | CEM1994045 | 28 | Male | White | 25 | 31 | high_anxiety | 72 | 60.0 | high_anxiety_(45-80) | -12 | 60 | 8 wk |

In [105…

```
import pandas as pd
import statsmodels.formula.api as smf
import plotly.express as px

# Read the CSV file
url = 'https://raw.githubusercontent.com/lauricocha/Digital-Art-Making-Research-2024/main/MFoA-data.csv'
MFOA = pd.read_csv(url)

# Display the first few rows of the dataframe to verify column names and data types
print(MFOA.head())
print(MFOA.dtypes)

# Ensure 'Weeks_Completed' is treated as a categorical variable
MFOA['Weeks_Completed'] = MFOA['Weeks_Completed'].astype('category')
```

```python
# Calculate 'STAI-change' as the difference between 'Last_Recorded_STAI' and 'STAI-T0'
MFOA['STAI-change'] = MFOA['Last_Recorded_STAI'] - MFOA['STAI-T0']

# Check for any missing values in critical columns and handle them if necessary
print(MFOA[['STAI-change', 'Weeks_Completed', 'VAIAK', 'SSCS']].isnull().sum())
```

```
           ID  Age  Gender Ethnicity ID  VAIAK  SSCS Initial_Anxiety_level  \
0  AMF1970039   52  Female      HispLat     53    39           low_anxiety
1  BNF2002007   20  Female        Asian     37    36           low_anxiety
2  NKM1972043   50    Male        Mixed     19    28      moderate_anxiety
3  CDF1998025   24  Female        White     21    23          high_anxiety
4  CEM1994045   28    Male        White     25    31          high_anxiety

   STAI-T0  STAI-T1       Final_Anxiety_Level  STAI-change  \
0       29     37.0       low_anxiety_(20-37)            8
1       25     23.0       low_anxiety_(20-37)           -2
2       41      NaN  moderate_anxiety_(38-44)           -1
3       52     47.0      high_anxiety_(45-80)           -5
4       72     60.0      high_anxiety_(45-80)          -12

   Last_Recorded_STAI Weeks_done  Weeks_Completed  Liking  Application
0                  37      8 wks                8    10.0         10.0
1                  23      8 wks                8     7.0          4.0
2                  40       1 wk                1     4.0          4.0
3                  47      8 wks                8     9.0         10.0
4                  60      8 wks                8     7.0          7.0
ID                        object
Age                        int64
Gender                    object
Ethnicity ID              object
VAIAK                      int64
SSCS                       int64
Initial_Anxiety_level     object
STAI-T0                    int64
STAI-T1                  float64
Final_Anxiety_Level       object
STAI-change                int64
Last_Recorded_STAI         int64
Weeks_done                object
Weeks_Completed            int64
Liking                   float64
Application              float64
dtype: object
STAI-change        0
Weeks_Completed    0
VAIAK              0
SSCS               0
dtype: int64
```

In [106…

```python
import pandas as pd
import statsmodels.formula.api as smf
import plotly.express as px

# Read the CSV file
url = 'https://raw.githubusercontent.com/lauricocha/Digital-Art-Making-Research-2024/main/MFoA-data.csv'
MFOA = pd.read_csv(url)

# Ensure 'Weeks_Completed' is treated as a categorical variable
MFOA['Weeks_Completed'] = MFOA['Weeks_Completed'].astype('category')

# Calculate 'STAI-change' as the difference between 'Last_Recorded_STAI' and 'STAI-T0'
MFOA['STAI_change'] = MFOA['Last_Recorded_STAI'] - MFOA['STAI-T0']

# Check for any missing values in critical columns and handle them if necessary
print(MFOA[['STAI_change', 'Weeks_Completed', 'VAIAK', 'SSCS']].isnull().sum())

# Drop rows with missing values in the required columns (if any)
MFOA = MFOA.dropna(subset=['STAI_change', 'Weeks_Completed', 'VAIAK', 'SSCS'])

# Print the first few rows to check column names
print(MFOA.head())

# Define the regression formula including interaction terms
formula = 'STAI_change ~ Weeks_Completed * VAIAK + Weeks_Completed * SSCS'

# Fit the model
model = smf.ols(formula, data=MFOA).fit()

# Print the summary of the model
print(model.summary())

# Visualizing the interaction effects
# Predicted values for visualization
MFOA['predicted'] = model.predict(MFOA)

# Create interaction plots for VAIAK and Weeks_Completed
fig = px.scatter(MFOA, x="VAIAK", y="predicted", color="Weeks_Completed",
                 size='Last_Recorded_STAI', hover_data=['VAIAK', 'SSCS', 'Weeks_Completed'])
fig.show()
```

```
STAI_change        0
Weeks_Completed    0
VAIAK              0
SSCS               0
dtype: int64
           ID  Age  Gender Ethnicity ID  VAIAK  SSCS Initial_Anxiety_level  \
0  AMF1970039   52  Female      HispLat     53    39           low_anxiety
1  BNF2002007   20  Female        Asian     37    36           low_anxiety
2  NKM1972043   50    Male        Mixed     19    28      moderate_anxiety
3  CDF1998025   24  Female        White     21    23          high_anxiety
4  CEM1994045   28    Male        White     25    31          high_anxiety

   STAI-T0  STAI-T1       Final_Anxiety_Level  STAI-change  \
0       29     37.0       low_anxiety_(20-37)            8
1       25     23.0       low_anxiety_(20-37)           -2
2       41      NaN  moderate_anxiety_(38-44)           -1
3       52     47.0      high_anxiety_(45-80)           -5
4       72     60.0      high_anxiety_(45-80)          -12

   Last_Recorded_STAI Weeks_done Weeks_Completed  Liking  Application  \
0                  37      8 wks               8    10.0         10.0
1                  23      8 wks               8     7.0          4.0
2                  40       1 wk               1     4.0          4.0
3                  47      8 wks               8     9.0         10.0
4                  60      8 wks               8     7.0          7.0

   STAI_change
0            8
1           -2
2           -1
3           -5
4          -12
                            OLS Regression Results
==============================================================================
Dep. Variable:            STAI_change   R-squared:                       0.579
Model:                            OLS   Adj. R-squared:                 -0.264
Method:                 Least Squares   F-statistic:                    0.6865
Date:                Sat, 22 Feb 2025   Prob (F-statistic):              0.740
Time:                        20:18:43   Log-Likelihood:                -63.175
No. Observations:                  22   AIC:                             156.3
Df Residuals:                       7   BIC:                             172.7
Df Model:                          14
Covariance Type:            nonrobust
================================================================================================
                                coef    std err          t      P>|t|      [0.025      0.975]
------------------------------------------------------------------------------------------------
Intercept                   -67.9571     51.480     -1.320      0.228    -189.688      53.774
Weeks_Completed[T.1]         70.2786     58.136      1.209      0.266     -67.191     207.748
Weeks_Completed[T.2]          0.9294      1.117      0.832      0.433      -1.713       3.571
Weeks_Completed[T.3]          0.0057      0.005      1.188      0.274      -0.006       0.017
Weeks_Completed[T.4]          0.0251      0.019      1.304      0.233      -0.020       0.071
Weeks_Completed[T.5]         -0.0090      0.034     -0.264      0.799      -0.090       0.072
Weeks_Completed[T.8]         64.3750     54.159      1.189      0.273     -63.691     192.441
VAIAK                         1.8857      1.104      1.708      0.131      -0.725       4.496
Weeks_Completed[T.1]:VAIAK   -2.0781      1.129     -1.841      0.108      -4.747       0.591
Weeks_Completed[T.2]:VAIAK   -4.2448      5.475     -0.775      0.464     -17.192       8.702
Weeks_Completed[T.3]:VAIAK    0.2412      0.203      1.188      0.274      -0.239       0.721
Weeks_Completed[T.4]:VAIAK    0.5014      0.384      1.304      0.233      -0.408       1.410
Weeks_Completed[T.5]:VAIAK   -0.3125      1.302     -0.240      0.817      -3.390       2.765
Weeks_Completed[T.8]:VAIAK   -1.3700      1.167     -1.174      0.279      -4.130       1.390
SSCS                         -0.8714      1.554     -0.561      0.592      -4.545       2.803
Weeks_Completed[T.1]:SSCS     0.8605      1.734      0.496      0.635      -3.239       4.960
Weeks_Completed[T.2]:SSCS     5.5786      6.475      0.862      0.417      -9.731      20.888
Weeks_Completed[T.3]:SSCS     0.2125      0.179      1.188      0.274      -0.211       0.636
Weeks_Completed[T.4]:SSCS     1.1030      0.846      1.304      0.233      -0.897       3.103
Weeks_Completed[T.5]:SSCS     0.9606      1.738      0.553      0.598      -3.148       5.069
Weeks_Completed[T.8]:SSCS     0.2780      1.685      0.165      0.874      -3.707       4.263
==============================================================================
Omnibus:                       21.421   Durbin-Watson:                   1.627
Prob(Omnibus):                  0.000   Jarque-Bera (JB):               32.395
Skew:                          -1.768   Prob(JB):                     9.24e-08
Kurtosis:                       7.779   Cond. No.                     3.65e+17
==============================================================================

Notes:
[1] Standard Errors assume that the covariance matrix of the errors is correctly specified.
[2] The smallest eigenvalue is 5.73e-31. This might indicate that there are
strong multicollinearity problems or that the design matrix is singular.
```

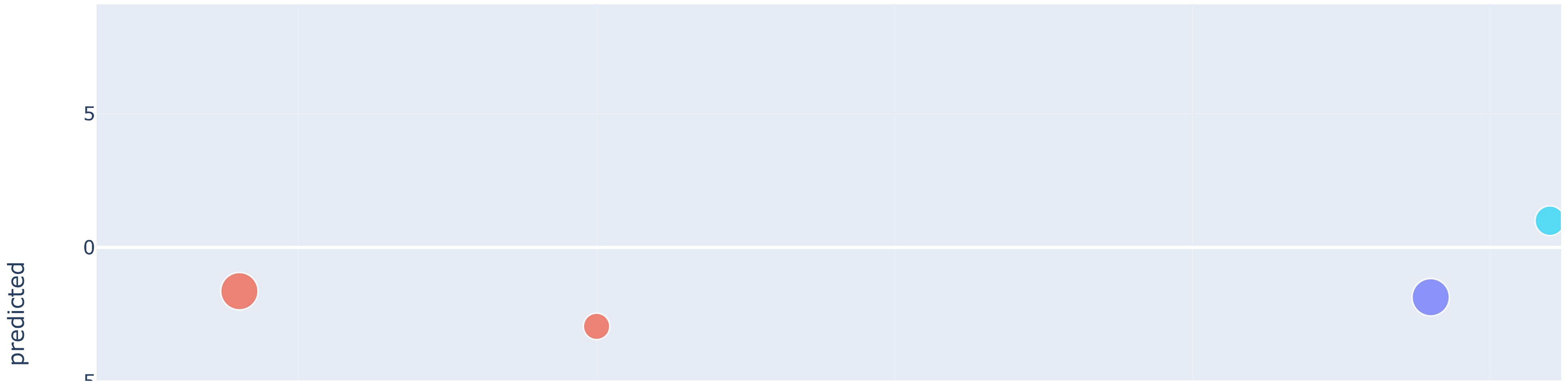


In [107…

```
import pandas as pd
import statsmodels.formula.api as smf
import plotly.express as px

# Read the CSV file
url = 'https://raw.githubusercontent.com/lauricocha/Digital-Art-Making-Research-2024/main/MFoA-data.csv'
MFOA = pd.read_csv(url)

# Ensure 'Weeks_Completed' is treated as a categorical variable
MFOA['Weeks_Completed'] = MFOA['Weeks_Completed'].astype('category')

# Calculate 'STAI-change' as the difference between 'Last_Recorded_STAI' and 'STAI-T0'
MFOA['STAI_change'] = MFOA['Last_Recorded_STAI'] - MFOA['STAI-T0']

# Check for any missing values in critical columns and handle them if necessary
print(MFOA[['STAI_change', 'Weeks_Completed', 'VAIAK', 'SSCS']].isnull().sum())

# Drop rows with missing values in the required columns (if any)
MFOA = MFOA.dropna(subset=['STAI_change', 'Weeks_Completed', 'VAIAK', 'SSCS'])

# Print the first few rows to check column names
print(MFOA.head())

# Define the regression formula including interaction terms
formula = 'STAI_change ~ Weeks_Completed * VAIAK + Weeks_Completed * SSCS'

# Fit the model
model = smf.ols(formula, data=MFOA).fit()

# Print the summary of the model
print(model.summary())

# Visualizing the interaction effects
# Predicted values for visualization
MFOA['predicted'] = model.predict(MFOA)

# Create interaction plots for VAIAK and Weeks_Completed
fig = px.scatter(MFOA, x="SSCS", y="predicted", color="Weeks_Completed",
                 size='Last_Recorded_STAI', hover_data=['VAIAK', 'SSCS', 'Weeks_Completed'])
fig.show()
```

```
STAI_change        0
Weeks_Completed    0
VAIAK              0
SSCS               0
dtype: int64
           ID  Age  Gender Ethnicity ID  VAIAK  SSCS Initial_Anxiety_level  \
0  AMF1970039   52  Female      HispLat     53    39           low_anxiety   
1  BNF2002007   20  Female        Asian     37    36           low_anxiety   
2  NKM1972043   50    Male        Mixed     19    28      moderate_anxiety   
3  CDF1998025   24  Female        White     21    23          high_anxiety   
4  CEM1994045   28    Male        White     25    31          high_anxiety   

   STAI-T0  STAI-T1       Final_Anxiety_Level  STAI-change  \
0       29     37.0       low_anxiety_(20-37)            8   
1       25     23.0       low_anxiety_(20-37)           -2   
2       41      NaN  moderate_anxiety_(38-44)           -1   
3       52     47.0      high_anxiety_(45-80)           -5   
4       72     60.0      high_anxiety_(45-80)          -12   

   Last_Recorded_STAI Weeks_done Weeks_Completed  Liking  Application  \
0                  37      8 wks               8    10.0         10.0   
1                  23      8 wks               8     7.0          4.0   
2                  40       1 wk               1     4.0          4.0   
3                  47      8 wks               8     9.0         10.0   
4                  60      8 wks               8     7.0          7.0   

   STAI_change  
0            8  
1           -2  
2           -1  
3           -5  
4          -12  
                            OLS Regression Results                            
==============================================================================
Dep. Variable:            STAI_change   R-squared:                       0.579
Model:                            OLS   Adj. R-squared:                 -0.264
Method:                 Least Squares   F-statistic:                    0.6865
Date:                Sat, 22 Feb 2025   Prob (F-statistic):              0.740
Time:                        20:18:43   Log-Likelihood:                -63.175
No. Observations:                  22   AIC:                             156.3
Df Residuals:                       7   BIC:                             172.7
Df Model:                          14                                         
Covariance Type:            nonrobust                                         
==============================================================================================
                                 coef    std err          t      P>|t|      [0.025      0.975]
----------------------------------------------------------------------------------------------
Intercept                    -67.9571     51.480     -1.320      0.228    -189.688      53.774
Weeks_Completed[T.1]          70.2786     58.136      1.209      0.266     -67.191     207.748
Weeks_Completed[T.2]           0.9294      1.117      0.832      0.433      -1.713       3.571
Weeks_Completed[T.3]           0.0057      0.005      1.188      0.274      -0.006       0.017
Weeks_Completed[T.4]           0.0251      0.019      1.304      0.233      -0.020       0.071
Weeks_Completed[T.5]          -0.0090      0.034     -0.264      0.799      -0.090       0.072
Weeks_Completed[T.8]          64.3750     54.159      1.189      0.273     -63.691     192.441
VAIAK                          1.8857      1.104      1.708      0.131      -0.725       4.496
Weeks_Completed[T.1]:VAIAK    -2.0781      1.129     -1.841      0.108      -4.747       0.591
Weeks_Completed[T.2]:VAIAK    -4.2448      5.475     -0.775      0.464     -17.192       8.702
Weeks_Completed[T.3]:VAIAK     0.2412      0.203      1.188      0.274      -0.239       0.721
Weeks_Completed[T.4]:VAIAK     0.5014      0.384      1.304      0.233      -0.408       1.410
Weeks_Completed[T.5]:VAIAK    -0.3125      1.302     -0.240      0.817      -3.390       2.765
Weeks_Completed[T.8]:VAIAK    -1.3700      1.167     -1.174      0.279      -4.130       1.390
SSCS                          -0.8714      1.554     -0.561      0.592      -4.545       2.803
Weeks_Completed[T.1]:SSCS      0.8605      1.734      0.496      0.635      -3.239       4.960
Weeks_Completed[T.2]:SSCS      5.5786      6.475      0.862      0.417      -9.731      20.888
Weeks_Completed[T.3]:SSCS      0.2125      0.179      1.188      0.274      -0.211       0.636
Weeks_Completed[T.4]:SSCS      1.1030      0.846      1.304      0.233      -0.897       3.103
Weeks_Completed[T.5]:SSCS      0.9606      1.738      0.553      0.598      -3.148       5.069
Weeks_Completed[T.8]:SSCS      0.2780      1.685      0.165      0.874      -3.707       4.263
==============================================================================
Omnibus:                       21.421   Durbin-Watson:                   1.627
Prob(Omnibus):                  0.000   Jarque-Bera (JB):               32.395
Skew:                          -1.768   Prob(JB):                     9.24e-08
Kurtosis:                       7.779   Cond. No.                     3.65e+17
==============================================================================

Notes:
[1] Standard Errors assume that the covariance matrix of the errors is correctly specified.
[2] The smallest eigenvalue is 5.73e-31. This might indicate that there are
strong multicollinearity problems or that the design matrix is singular.
```

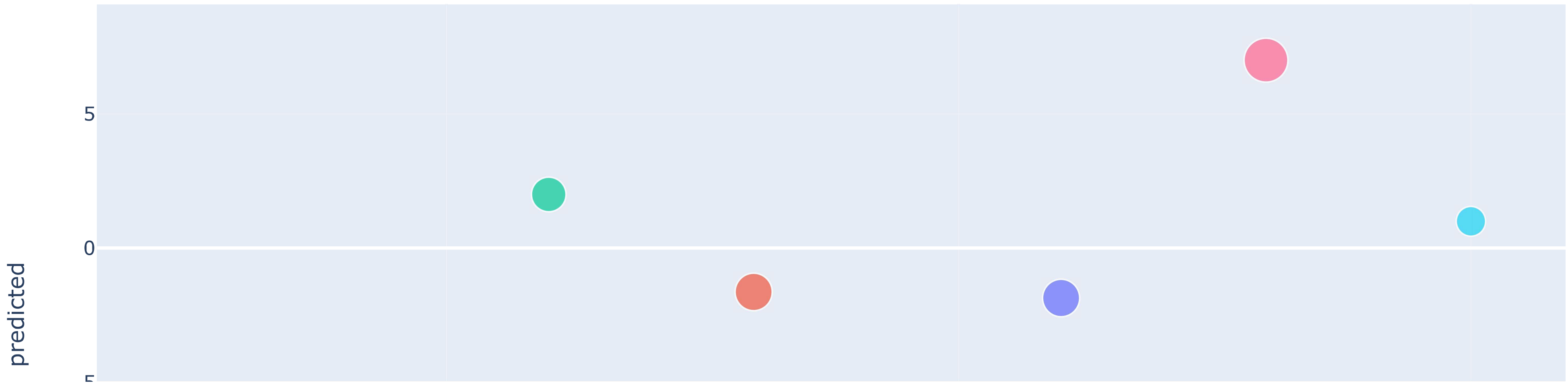


## Scatter plots to illustrate the relationship betweenSSCSK scores and change in STAI level

In [109…

```
import plotly.express as px
import pandas as pd
import plotly.io as pio
from IPython.display import Image

# Read the CSV file
url = 'https://raw.githubusercontent.com/lauricocha/Digital-Art-Making-Research-2024/main/MFoA-data.csv'
df = pd.read_csv(url)

# Create a scatter plot
fig = px.scatter(df, x="SSCS", y="STAI-T0", color="Initial_Anxiety_level",
                 size='STAI-T0', hover_data=['SSCS'])

# Update layout
fig.update_layout(
    height=600,
    width=490,
    title=dict(
        text="Initial STAI score",
        x=0.5,  # Center the title horizontally
        xanchor='center',
        yanchor='top'
    ),
    title_font=dict(size=22)
)

# Save the figure as a static image using kaleido
pio.write_image(fig, "plotly_figure_initial.png")

# Display the static image
display(Image("plotly_figure_initial.png"))
```

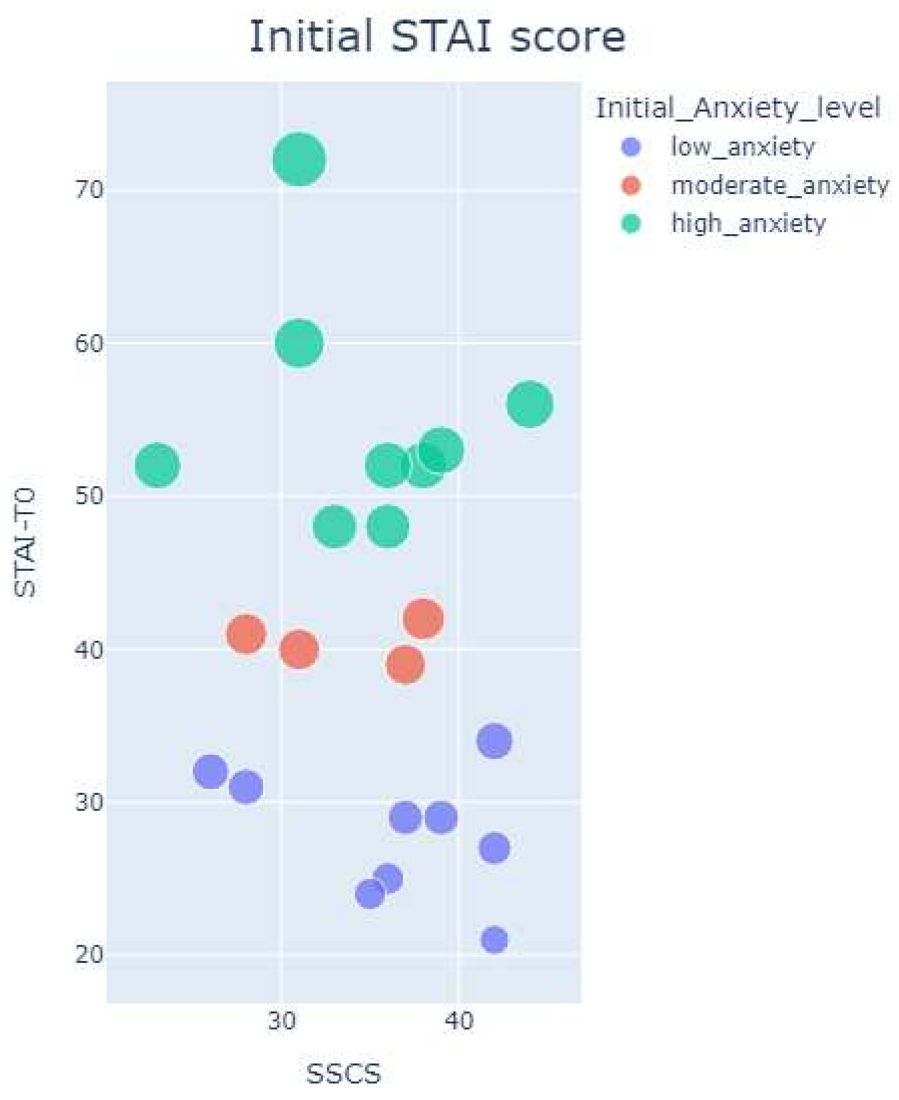


In [110…

```
import plotly.express as px
import pandas as pd
import plotly.io as pio
from IPython.display import Image

# Read the CSV file
url = 'https://raw.githubusercontent.com/lauricocha/Digital-Art-Making-Research-2024/main/MFoA-data.csv'
df = pd.read_csv(url)

# Create a scatter plot
fig = px.scatter(df, x="SSCS", y="Last_Recorded_STAI", color="Final_Anxiety_Level",
                 size='Last_Recorded_STAI', hover_data=['SSCS'])

# Update the y-axis range
fig.update_yaxes(range=[15, 80])

# Update layout
fig.update_layout(
    height=600,
    width=630,
    showlegend=True,
    title=dict(
        text="Final STAI score",
        x=0.4,  # Center the title horizontally
        xanchor='center',
        yanchor='top'
    ),
    title_font=dict(size=22)
)

# Save the figure as a static image using kaleido
pio.write_image(fig, "plotly_figure_final_stai.png")

# Display the static image
display(Image("plotly_figure_final_stai.png"))
```

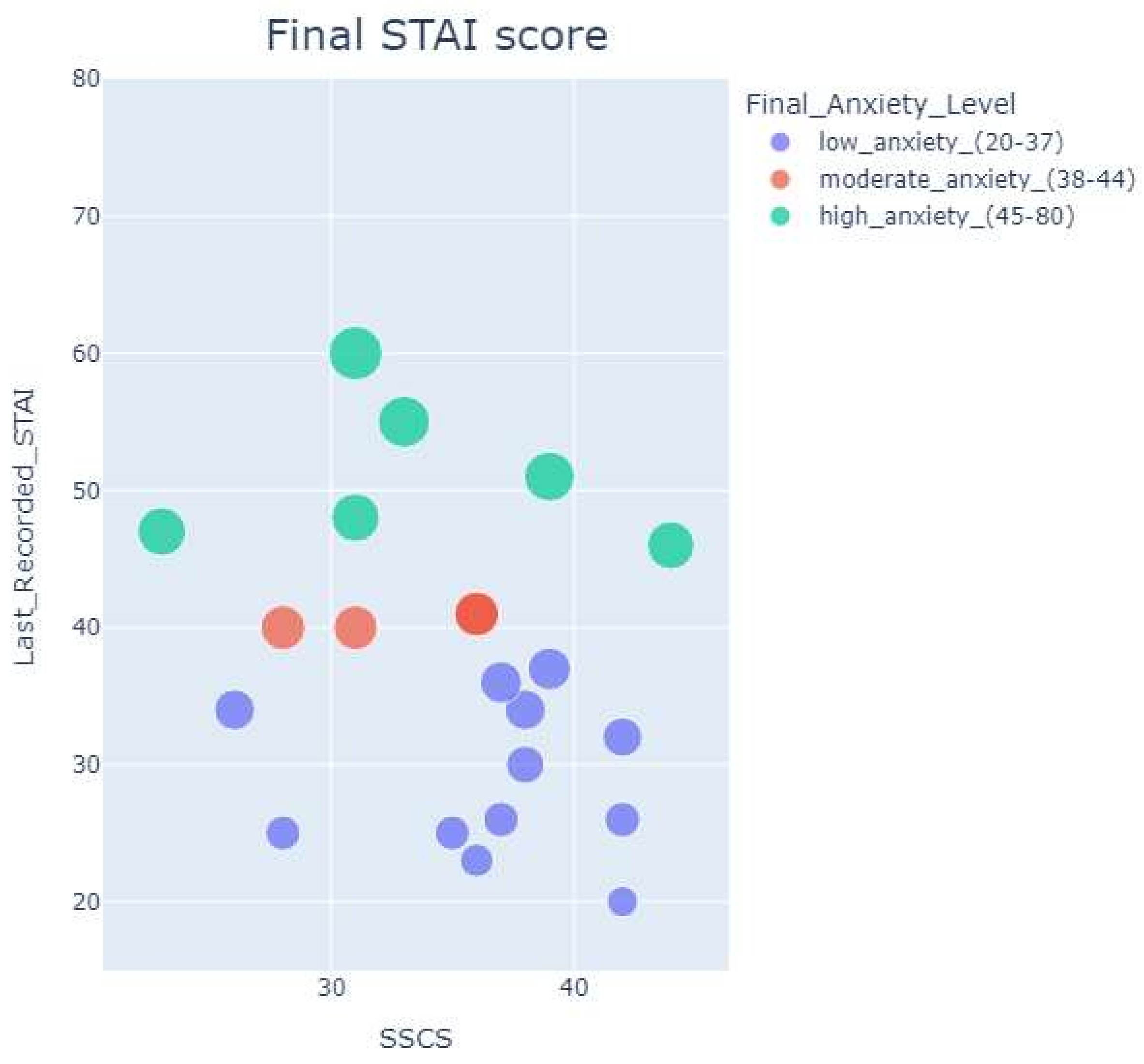
Final STAI score
Last_Recorded_STAI
SSCS
80
70
60
50
40
30
20
30
40
Final_Anxiety_Level
low_anxiety_(20-37)
moderate_anxiety_(38-44)
high_anxiety_(45-80)

In [ ]:
